# Bioinformatics and expression analysis of histone modification genes in grapevine predict their involvement in seed development, powdery mildew resistance, and hormonal signaling

**DOI:** 10.1186/s12870-020-02618-7

**Published:** 2020-09-04

**Authors:** Li Wang, Bilal Ahmad, Chen Liang, Xiaoxin Shi, Ruyi Sun, Songlin Zhang, Guoqiang Du

**Affiliations:** 1 College of Horticulture, Hebei Agricultural University, Baoding, 071000 Hebei China; 2 State Key Laboratory of Crop Stress Biology in Arid Areas, College of Horticulture, Northwest A&F University, Yangling, 712100 Shaanxi China

**Keywords:** *VvHMs*, *VvSDG*, Seed development, Phytohormones, Evolutionary history

## Abstract

**Background:**

Histone modification genes (HMs) play potential roles in plant growth and development via influencing gene expression and chromatin structure. However, limited information is available about HMs genes in grapes (*Vitis vinifera* L.).

**Results:**

Here, we described detailed genome-wide identification of HMs gene families in grapevine. We identified 117 HMs genes in grapevine and classified these genes into 11 subfamilies based on conserved domains and phylogenetic relationships with Arabidopsis. We described the genes in terms of their chromosomal locations and exon-intron distribution. Further, we investigated the evolutionary history, gene ontology (GO) analysis, and syntenic relationships between grapes and Arabidopsis. According to results 21% HMs genes are the result of duplication (tandem and segmental) events and all the duplicated genes have negative mode of selection. GO analysis predicted the presence of HMs proteins in cytoplasm, nucleus, and intracellular organelles. According to seed development expression profiling, many HMs grapevine genes were differentially expressed in seeded and seedless cultivars, suggesting their roles in seed development. Moreover, we checked the response of HMs genes against powdery mildew infection at different time points. Results have suggested the involvement of some genes in disease resistance regulation mechanism. Furthermore, the expression profiles of HMs genes were analyzed in response to different plant hormones (Abscisic acid, Jasmonic acid, Salicylic acid, and Ethylene) at different time points. All of the genes showed differential expression against one or more hormones.

**Conclusion:**

*VvHMs* genes might have potential roles in grapevine including seed development, disease resistance, and hormonal signaling pathways. Our study provides first detailed genome-wide identification and expression profiling of HMs genes in grapevine.

## Background

There are several posttranscriptional modification systems and processes including methylation, acetylation, phosphorylation, ubiquitination, and sumoylation, which can influence gene expression [1]. Histone modification and DNA methylation are very important because they regulate the pattern of gene expression by altering DNA availability and chromatin structure. These mechanisms are necessary in plant life cycle for the normal development and disease resistance [2]. Acetylation, deacetylation, methylation, and demethylation are common histone modifications (HMs) processes. These HMs processes are mostly controlled by four different histone modification gene families, which include histone methyltransferases (HMTs), histone methylases (HDMs), histone acetylases (HATs), and histone deacetylases (HDACs) [3, 4].

These HMs gene families HMTs, HDMs, HATs, and HDACs contain 2, 2, 4, and 3 subfamilies, respectively. Each subfamily has specific domain or structure, thereby, plays different role in plant growth and development. Set domain group (SDG) and protein arginine methyltransferases (PRMTs) are subfamilies of HMTs, and contain set domain and Prma domain, respectively. The HDMs have HDMA (SWIRN and C-terminal domain) and JMJ (JmjC domain-containing proteins) subfamily. In *Arabidopsis thaliana*, SDG proteins have been divided into seven classes on the basis of sequence similarity with set domain. The classes are I), Enhancer of zesta, II) ASH1 homologs, III) Trx homologs and related proteins, IV) Proteins having SET and PHD domain, V), Su (var) homologs, VI) Proteins having interrupted SET domain (S-ET), and VII) Ribulose-1, 5-bisphosphate carboxylase/oxygenase (Rubisco) methyltransferase (RBCMT). Members of every class have specific structures and functions [5–7]. According to the sequence characterization and in silico analysis of data, HATs are divided into four subfamilies: (1) HAC have p300/CREB (cAMP responsive element-binding protein)-binding protein (CBP) family), (2) HAG contained the general control non repressible 5-related N-terminal acetyltransferase (GNAT) family, (3) HAF contained the TATA-binding protein-associated factor (TAF_II_250) family, and (4) HAM type had MOZ, Ybf2/Sas3, Sas2, and Tip60 (MYST) family. Furthermore, HDACs are divided into HDA (RPD3 (Reduced Potassium Dependence) /HDA1, SRT (Silent information regulator 2, SIR2), and HDT (HD2 ) family [8, 9].

Methylation is an important gene regulation mechanism, which can affect different processes and have potential roles in plant growth and development. Histone lysine methylation modifications are mainly catalyzed by different groups of histone methyltransferases (HMTs). Mostly, SDG genes play role in histone lysine methylation modifications. CURLY LEAF (CLF) and MEDEA (MEA) are the first recognized members of SDG [10, 11]. HMTs play multiple roles in plant life cycle such as cell cycle regulation, floral organ development, flowering transition, transcriptional activation and silencing, regulation of dormancy, fungal pathogens resistance, and influence on hormone signaling pathway genes during different (biotic & abiotic) stresses [12, 13]. For example, *AtASHH2* methylated H3K4 was recognized as a positive regulator of gene expression. Some SDG genes play potential roles in heterochromatin formation and gene silencing [14]. *AtSDG5* has been reported for its involvement in embryo abortion and endosperm development. *AtSDG2* influences gametophyte and sporophyte development [11]. PRMT proteins also play different roles in plants such as AtPRMT4A and 4B collectively influenced flowering time in Arabidopsis. In Arabidopsis, mutations in PRMT5 resulted in dark green and curly leaves with stunted growth [15].

HATs and HDACs play critical roles in the regulation of gene expression in plant growth and plant response to different stresses. For example, *AtHAM1* and *AtHAM2* have role in male and female gamete formation [16]. Silencing of *AtHD2A* expression resulted in aborted seed development [17]. Furthermore, *AtHDA7* and *AtHD2B* have role in embryo development and seed dormancy, respectively [18, 19]. Overexpression of *OsglHAT1* increased grain weight and plant biomass by increasing cell number and rate of grain filling [20]. Functional analysis of *AtHDA15* and *AtHDA19* have shown their involvement in phtomorphogenesis [21, 22]. In other studies, *AtHDA19* has shown involvement in leaf and root development. These results suggest that HDA genes have varying roles in plants. *AtHDA5* and *AtHDA9* can regulate flowering time. Moreover, some genes such as *OsHAC701*, *OsHAG703*, *AtHD2C*, and *OsHDT701* have been reported for temperature, salt, and abscisic acid (ABA) stress response [23–26]. *AtHDA19* is involved in plant defense against fungal and bacterial diseases via influencing the expression of JA, SA, and ethylene signaling pathway genes. Finally, HATs and HDACs play potential roles in transcriptional regulation of many developmental processes via interacting with transcription factors and various chromatin-remodeling factors [3].

Different families of HMs genes have been identified in many plant species including, *A. thaliana*, *Oryza sativa*, *Solanum lycopersicum*, *Malus domestica*, *Citrus sinensis*, *Fragaria vesca*, *Zea mays*, and *Brassica rapa*. However, all four HMs gene families have been described only in *A. thaliana, S. lycopersicum, M. domestica*, and *C. sinensis* [18, 27–32]*.* Moreover, most of the functional studies have been carried out only in model plants e.g. Arabidopsis and rice. There is little information about the HMs genes number and functions in grapevine. The potential roles of HMs genes in different plants during different stages of plant growth and involvement in defense mechanism, justifies the need of comprehensive bioinformatics and expression analysis of HMs genes in grapevine. To the best of our knowledge this is the first comprehensive bioinformatic analysis of all four HMs gene families in grapevine. In this study, we performed identification of HMs genes in grapevine and then divided into different families on the basis of phylogenetic analysis with Arabidopsis. Moreover, carried out their exon-intron, evolutionary, synteny, and gene ontology (GO) analysis. We also analyzed the response of HMs genes against powdery mildew infection and under different hormone treatments, along with expression profiling during different stages of seed development in seeded and seedless cultivars. This study will increase understandings about grapevine HMs genes and will provide assistance for functional characterization of genes.

## Methods

### Identification of HMs genes in grapevine

To identify a complete list of HMs genes in the grapevine, Hidden Markov Model (HMM) profile of each gene family was downloaded from Pfam database (http://pfam.xfam.org/search#tabview=tab1) [30]. These HMM profiles were used as query against three public databases: Grape Genome Database (http://www.genoscope.cns.fr), the National Centre for Biotechnology Information (http://www.ncbi.nlm.nih.gov/), and the Grapevine Genome CRIBI Biotech website (http://genomes.cribi.unipd.it/) by HMMER software to find full list of HMs proteins in grapevine [33]. Moreover, for the identification of HDT family, Basic Local Alignment Search Tool (BLAST) was used because this family has no HMM profile in Pfam database [30]. Previously, identified Arabidopsis and apple proteins annotated as HDT were taken and used as query for BlastP. Initially 148 HMs genes were identified. All non-redundant putative HMs genes were checked for conserved domains using SMART (http://smart.embl-heidelberg.de), and genes having incomplete domain or DUF (Domain of unknown function) were removed. In this way, 117 *VvHMs* genes were selected for final consideration.

### Phylogenetic and exon- intron distribution analysis of HMs genes

The HMs protein sequences of Arabidopsis and grapes were used for phylogenetic analysis. MEGA 6.0 software was used to construct phylogenetic trees with the following parameters: ‘W’ approach for sequence alignments, the neighbor-joining (NJ) method, p-distance, complete deletion and 1000 bootstrap values [34]. Different phylogenetic trees were generated for different subfamilies. For generation of phylogenetic trees among *VvHMS* genes of same group, we followed the previous trend. Phylogenetic trees were constructed only among members of three well studied subfamilies. The full genomic sequences and coding sequences of grapes were downloaded from Grape Genome Database (http://www.genoscope.cns.fr) and online Gene Structure Display Server 2.071 (http://gsds.cbi.pku.edu.cn/index.php) was used for exon-intron analysis.

### Synteny analysis of grapevine and Arabidopsis genes

Two or more genes on the same chromosome within 200 kb were designated as tandemly duplicated [35]. The list of synteny region pairs within grape genome and comparison of duplication events between grape and Arabidopsis were downloaded from Plant Genome Duplication Database (http://chibba.agtec.uga.edu/duplication/) [36]. Circos version 0.63 (http://circos.ca/) with default parameters was used for generation of diagrams.

### Estimation of Ka/Ks ratio for duplicated genes and Go analysis

An online software (http://services.cbu.uib.no/tools/kaks) was used for calculation of Non-synonymous substitution rate (Ka) and synonymous substitution rate (Ks) of duplicated genes. The Ka/Ks ratio was used for the estimation of selection pressure. The Ka/Ks value denotes three different situations of selection pressure i.e. neutral (equal to 1), positive (more than 1), and negative (less than 1) [37]. GO enrichment analysis was performed using MetGenMAP with default parameters [38].

### Plant materials

In this study, five grapevine cultivars were used including two seeded cultivars (‘Kyoho’ and ‘Muscat kyoho’), two seedless cultivars (‘Flame seedless’ and ‘Crimson seedless’), and one powdery mildew-resistant, Chinese wild cultivar ‘Shang-24’ (*V. quinquangularis*). All the cultivars were maintained under natural environmental conditions at the grapevine orchard of Hebei Agricultural University, Baoding, Hebei, China (38°51′N, 115°29′E). Where the average annual temperature is 13.4 °C, (Below - 4.3 °C in winter and above 26.4 °C in summer), the average annual sunshine hours are 2511, and the mean annual precipitation is 498.9 mm. Young leaves of ‘Shang-24’ were inoculated with *Erysiphe necator* (Schw.) and harvested at 12, 24, and 48 h post-inoculation. Control leaves were inoculated with sterile water. For the preparation of hormone solution, ABA, Methyl jasmonate (MeJA), and Salicylic acid (SA) were first dissolved in 95% ethanol and then diluted down with water, while ethylene was directly dissolved in water. Hormone treatments were performed as a foliar spray with 300 μM ABA, 0.5 g/L ethylene, 50 μM MeJA or 100 μM SA, and samples were collected at 1, 6, and 12 h post-treatment. Control leaves were sprayed with sterile water. Furthermore, for seed development studies seeds were dissected from seedless and seeded cultivars at 20, 30, 40, and 50 days after flowering (DAF). All leaves and seed samples were immediately frozen in liquid nitrogen and stored at − 80 °C for future use. All the plant materials used and collected in this work comply with China’s guidelines and legislation.

### Transcriptome analysis of *VvHMs* genes during seed development in seeded and seedless grape progenies

Expression profiles of *VvHMs* genes during seed development from seeded and seedless progenies were analyzed using our previously published transcriptome data (BioProject Accession, PRJNA338255) [39]. Cross-progeny populations from the seeded maternal parent ‘Red Globe’ and the seedless paternal parent ‘Centennial seedless’ were used. A total of 65 progeny were obtained including 31 seedless and 34 seeded. Nine seedless progeny and 9 seeded ones were randomly selected from the populations and were used for RNA-sequencing. Reads per kilobase of exon model per million mapped reads (RPKM) of *VvHMs* were transformed into heat maps using Mev 4.8.1 [40]. RPKM value of each VvHM gene was normalized based on the mean RPKM value of genes in all samples analyzed. Further, processed data was log2 transformed and was used to generate heatmap with Mev 4.8.1. Red and green color scale indicate high and low expression levels, respectively.

### RNA extraction and expression analysis by quantitative RT-PCR

Total RNA was extracted from samples by using the EZNA Plant RNA Kit (R6827–01, OMEGA Biotek, Norcross, GA, USA) according to manufacturer’s guidelines. Nano drop spectrophotometer (Thermo Fisher Scientific, Yokohama, Japan) was used to measure OD value of RNA, and each RNA sample was subjected to agarose gel electrophoresis to check its quality. The Prime Script RTase (Trans Gen Biotech, Beijing, China) was used to remove gDNA and then for reverse transcription. Genome DNA was removed by gDNA Eraser (contains DNase). A qPCR reaction on crude RNA was also performed in order to access the degree of gDNA contamination. After this cDNA was diluted six times with distilled water for a final concentration of 100 ng/μl. The grape *ACTIN* gene (GenBank Accession number NC_012010) and *EF1-α* gene (GenBank Accession number NC_012012) were used as internal reference genes. All primers were designed using Primer 5 software and can be found in the Additional file 1: Table S1. Three biological replicates and three technical replicates were used. For each reaction, 100 ng cDNA was used and carried out in triplicate with a reaction volume of 20 μl containing 0.8 μl each primer (1.0 μM), 1.0 μl of cDNA, 10 μl of 2 X Fast Super EvaGreen® qPCR Mastermix (US Everbright Inc., Suzhou, China), and 7.4 μl sterile distilled water. The amplification parameters were 95 °C for 30 s, followed by 40 cycles of 95 °C for 5 s and 60 °C for 30 s. Melting-curve analyses were performed with initial incubation at 95 °C for 15 s and then a constant increase from 60 to 95 °C. Relative expression levels were analyzed using the IQ5 software and the geNorm software.

### Statistical analysis

Data was analyzed using SPSS Statistics 22.0 software. ANOVA with a Tukey post-hoc analysis was used to check the overall significance of data. Sigma Plot 12 was used to draw the graphs.

## Results

### Genome-wide identification and annotation of HMs genes

A total of 117 putative HMs genes were identified in the grape genome having 48 HMTs, 22 HDMs, 33 HATs, and 14 HDACs genes. According to previous studies [30–32], all of the genes were divided into 11 subfamilies based on the conserved domains. HMTs have 43 SDG and 5 PRMT genes; HDMs contain 15 JMJ and 7 HDMA genes; HATs have 28 HAG, 2 HAM, 2 HAC, and 1 HAF gene; and HDACs have 10, 2, and 2 genes of HDA, SRT, and HDT, respectively. These genes were named according to their families and their position on chromosomes. The predicted length of proteins in the SDG, PRMT, JMJ, HDMA, HAG, and HDA subfamilies ranges from 184 to 2199, 337–651, 131–1905, 492–984, 157–667, and 349–698 amino acids, respectively. Detailed information about genes including accession number, gene ID, chromosomal position, length of coding sequence, and length of protein sequence are presented in Table 1.

**Table 1:** List of *VvHM* genes

Gene Name	Accession No.	VCost.v3 ID	Chromosome	Start	End	CDS (bp)	ORF (aa)
**SDG gene family**							
*VvSDG1*	XP_019077603.1	Vitvi01g00086	1	949,657	957,893	1449	482
*VvSDG2*	XP_002281246.1	Vitvi01g00484	1	5,390,829	5,401,654	1452	484
*VvSDG3*	XP_002267249.1	Vitvi01g01058	1	14,007,416	14,010,547	1455	485
*VvSDG4*	XP_019078100.1	Vitvi01g01765	1	22,469,075	22,482,650	1458	486
*VvSDG5*	XP_010657698.1	Vitvi01g01812	1	22,873,387	22,877,304	984	328
*VvSDG6*	XP_010648716.1	Vitvi04g00550	4	5,937,871	5,970,124	3336	1111
*VvSDG7*	XP_019075140.1	Vitvi04g00878	4	11,443,796	11,463,685	1350	449
*VvSDG8*	XP_019075187.1	Vitvi04g01029	4	15,011,615	15,039,726	1698	565
*VvSDG9*	XP_002269759.2	Vitvi04g01208	4	17,398,366	17,416,188	3948	1316
*VvSDG10*	XP_002281774.1	Vitvi04g01653	4	22,226,309	22,252,207	1125	375
*VvSDG11*	XP_002282057.2	Vitvi05g00821	5	9,227,788	9,230,583	1554	518
*VvSDG12*	XP_002282386.1	Vitvi05g00855	5	9,819,374	9,821,392	1767	588
*VvSDG13*	XP_002268621.1	Vitvi05g02135	5	20,741,174	20,798,303	3207	1068
*VvSDG14*	XP_010652245.1	Vitvi07g00417	7	4,010,241	4,015,186	1149	382
*VvSDG15*	XP_019076867.1	Vitvi07g00675	7	7,299,138	7,313,963	2148	715
*VvSDG16*	XP_019076670.1	Vitvi07g01721	7	16,552,767	16,572,286	2805	935
*VvSDG17*	XP_002267602.2	Vitvi07g02035	7	20,552,339	20,557,870	1503	500
*VvSDG18*	XP_002277066.1	Vitvi08g00145	8	2,654,151	2,659,935	555	184
*VvSDG19*	XP_002278728.1	Vitvi08g00900	8	11,294,684	11,297,803	1434	478
*VvSDG20*	XP_002276611.1	Vitvi08g01786	8	20,728,208	20,735,141	1983	661
*VvSDG21*	XP_010655344.2	Vitvi10g00002	10	36,174	37,778	1512	504
*VvSDG22*	XP_002272781.1	Vitvi11g00800	11	9,538,580	9,561,355	1485	494
*VvSDG23*	XP_010656836.1	Vitvi11g01240	11	18,553,723	18,566,361	1752	584
*VvSDG24*	XP_002274324.1	Vitvi12g00537	12	7,248,181	7,258,220	1395	465
*VvSDG25*	XP_010657340.1	Vitvi12g00568	12	7,477,102	7,493,792	6600	2199
*VvSDG26*	XP_002267469.2	Vitvi12g00746	12	10,000,712	10,005,486	1494	497
*VvSDG27*	XP_010658301.1	Vitvi13g00002	13	12,083	63,791	2151	717
*VvSDG28*	XP_002275729.1	Vitvi13g00215	13	2,012,817	2,037,101	1464	487
*VvSDG29*	XP_010660173.1	Vitvi14g00738	14	12,583,680	12,614,451	582	193
*VvSDG30*	XP_010660678.1	Vitvi14g01462	14	24,836,624	24,881,775	2106	702
*VvSDG31*	XP_010661928.1	Vitvi15g00976	15	17,677,390	17,689,653	3012	1004
*VvSDG32*	XP_019081670.1	Vitvi16g00036	16	370,309	399,658	1317	438
*VvSDG33*	XP_002277774.1	Vitvi16g00290	16	5,345,035	5,346,423	1332	443
*VvSDG34*	XP_019081389.1	Vitvi16g00314	16	5,891,859	5,894,009	1671	557
*VvSDG35*	XP_010662976.1	Vitvi16g01288	16	20,769,710	20,783,861	3057	1019
*VvSDG36*	XP_002275757.1	Vitvi16g02079	16	21,677,420	21,681,935	1203	401
*VvSDG37*	XP_010664163.1	Vitvi18g00220	18	2,259,998	2,287,308	5877	1959
*VvSDG38*	XP_010664254.1	Vitvi18g00318	18	3,446,050	3,490,947	1433	481
*VvSDG39*	XP_019072296.1	Vitvi18g02964	18	19,242,564	19,274,978	2589	863
*VvSDG40*	XP_003634538.1	Vitvi18g02965	18	19,280,780	19,290,355	1113	370
*VvSDG41*	XP_010665141.1	Vitvi18g01629	18	19,587,111	19,592,826	1602	534
*VvSDG42*	XP_010646790.1	Vitvi02g01790	Un	22,578,033	22,580,210	1581	526
*VvSDG43*	XP_019074011.1	Vitvi10g00297	Un	28,765,455	28,776,585	3263	1127
**PRMT gene family**							
*VvPRMT1*	XP_019075123.1	Vitvi04g00275	4	2,582,427	2,595,604	1953	651
*VvPRMT2*	XP_002273923.1	Vitvi05g00322	5	3,203,018	3,217,695	1578	525
*VvPRMT3*	XP_002267286.2	Vitvi10g01540	10	17,242,398	17,277,211	1245	414
*VvPRMT4*	XP_002282760.1	Vitvi11g00208	11	2,016,637	2,022,471	1053	351
*VvPRMT5*	XP_002285026.1	Vitvi11g00426	11	4,126,320	4,146,088	1014	337
**JMJ gene family**							
*VvJMJ1*	XP_010660757.1	Vitvi02g00329	2	3,074,405	3,119,122	4488	1495
*VvJMJ2*	XP_010663122.1	Vitvi02g00759	2	8,573,734	8,582,220	2199	732
*VvJMJ3*	XP_010648867.1	Vitvi04g00769	4	9,601,201	9,680,561	2589	862
*VvJMJ4*	XP_010652382.1	Vitvi07g00618	7	6,483,761	6,483,761	2364	787
*VvJMJ5*	XP_002269129.1	Vitvi08g00216	8	4,253,675	4,259,819	393	131
*VvJMJ6*	XP_010655372.1	Vitvi10g00053	10	462,316	466,886	1941	646
*VvJMJ7*	XP_010655862.1	Vitvi10g01120	10	11,527,946	11,538,707	2856	952
*VvJMJ8*	XP_010655918.1	Vitvi10g01394	10	14,142,558	14,157,354	3060	1019
*VvJMJ9*	XP_010658392.1	Vitvi13g00630	13	5,944,228	5,969,863	4653	1551
*VvJMJ10*	XP_010659626.1	Vitvi14g00196	14	2,053,704	2,057,478	1473	490
*VvJMJ11*	XP_002266063.2	Vitvi14g00627	14	10,528,574	10,546,325	3537	1178
*VvJMJ12*	XP_019081261.1	Vitvi15g00950	15	17,348,763	17,355,815	2694	898
*VvJMJ13*	XP_002272599.2	Vitvi16g00120	16	1,798,809	1,811,815	3030	1009
*VvJMJ14*	XP_010662857.1	Vitvi16g01410	16	21,915,589	21,926,695	5987	1905
*VvJMJ15*	XP_010663055.1	Vitvi17g01001	17	11,978,867	11,993,275	2850	950
**HDMA gene family**							
*VvHDMA1*	XP_003631607.1	Vitvi03g00349	3	5,641,165	5,655,774	2952	984
*VvHDMA2*	XP_002265069.1	Vitvi03g01141	3	16,377,478	16,379,930	2010	669
*VvHDMA3*	XP_010658366.1	Vitvi13g00661	13	6,501,373	6,505,907	2805	935
*VvHDMA4*	XP_002280389.1	Vitvi17g00081	17	717,752	722,776	1692	563
*VvHDMA5*	XP_002275451.1	Vitvi18g00045	18	531,976	535,425	1479	492
*VvHDMA6*	XP_010664183.1	Vitvi18g00243	18	2,543,733	2,568,290	2028	675
*VvHDMA7*	XP_010664765.1	Vitvi18g01059	18	11,641,452	11,647,885	2316	772
**HAG gene family**							
*VvHAG1*	XP_002284766.1	Vitvi04g00414	4	4,185,433	4,190,152	495	165
*VvHAG2*	XP_002272537.1	Vitvi05g00123	5	788,238	792,303	1254	417
*VvHAG3*	XP_002278864.1	Vitvi05g00523	5	5,399,371	5,399,889	519	172
*VvHAG4*	XP_002282836.1	Vitvi05g00524	5	5,401,210	5,402,158	543	180
*VvHAG5*	XP_002278620.1	Vitvi06g00679	6	7,667,663	7,669,004	1155	384
*VvHAG6*	XP_010651587.1	Vitvi06g01092	6	14,154,983	14,172,657	867	289
*VvHAG7*	XP_002275146.2	Vitvi07g00052	7	77,994	88,805	1251	417
*VvHAG8*	XP_010651956.1	Vitvi07g00050	7	89,628	110,743	1317	439
*VvHAG9*	XP_002264391.3	Vitvi17g00723	7	948,363	958,053	1023	341
*VvHAG10*	XP_002278246.1	Vitvi07g00126	7	1,190,489	1,221,867	525	175
*VvHAG11*	XP_010652717.1	Vitvi07g01604	7	15,521,919	15,523,764	1266	422
*VvHAG12*	XP_010653125.1	Vitvi08g00051	8	875,613	882,448	666	222
*VvHAG13*	XP_010654086.1	Vitvi08g01460	8	17,298,621	17,301,373	474	157
*VvHAG14*	XP_019077012.1	Vitvi08g01691	8	19,763,293	19,771,775	2001	667
*VvHAG15*	XP_002264353.1	Vitvi09g01127	9	16,169,584	16,171,633	1203	400
*VvHAG16*	XP_002278105.2	Vitvi11g00083	11	928,669	934,564	1572	524
*VvHAG17*	XP_002270456.1	Vitvi11g00847	11	10,597,926	10,613,592	840	280
*VvHAG18*	XP_002267085.1	Vitvi12g00340	12	5,074,039	5,075,368	477	159
*VvHAG19*	XP_010657419.1	Vitvi13g01304	12	9,037,307	9,044,911	588	196
*VvHAG20*	XP_002270840.1	Vitvi13g01879	13	101,740	106,579	825	275
*VvHAG21*	XP_002273746.1	Vitvi13g00612	13	5,707,150	5,718,261	813	271
*VvHAG22*	XP_002273620.1	Vitvi13g00635	13	6,006,136	6,007,252	663	221
*VvHAG23*	XP_002273592.1	Vitvi13g01641	13	21,451,060	21,451,860	483	160
*VvHAG24*	XP_002276364.1	Vitvi14g01459	14	24,820,195	24,822,192	615	205
*VvHAG25*	XP_002273780.1	Vitvi16g00111	16	1,730,687	1,738,891	765	255
*VvHAG26*	XP_002262701.1	Vitvi17g01026	17	12,379,862	12,388,649	1368	455
*VvHAG27*	XP_019073388.1	Vitvi17g01073	17	390,224	405,802	930	310
*VvHAG28*	XP_019071767.1	Vitvi18g02063	18	25,838,001	25,839,742	864	288
**HAM gene family**							
*VvHAM1*	XP_002282931.1	Vitvi11g00219	11	2,093,896	2,100,497	1536	512
*VvHAM2*	XP_002285829.1	Vitvi18g00333	18	3,700,491	3,718,099	1140	379
**HAC gene family**							
*VvHAC1*	XP_019080308.1	Vitvi14g01230	9	22,849,657	22,861,791	3894	1297
*VvHAC2*	XP_019080308.1	Vitvi14g01230	14	21,859,980	21,865,827	1974	658
**HAF gene family**							
*VvHAF1*	XP_010656962.1	Vitvi12g00328	12	4,766,938	4,820,598	5397	1799
**HDA gene family**							
*VvHDA1*	XP_002283371.1	Vitvi03g00273	3	3,061,225	3,067,402	1185	394
*VvHDA2*	XP_002277742.1	Vitvi04g00077	4	781,102	787,349	1080	360
*VvHDA3*	XP_019075100.1	Vitvi04g01715	4	23,072,108	23,108,432	1314	437
*VvHDA4*	XP_010651716.1	Vitvi06g01425	6	19,410,537	19,425,212	1293	431
*VvHDA5*	XP_002277237.1	Vitvi06g01465	6	20,001,869	20,006,704	1047	349
*VvHDA6*	XP_019080589.1	Vitvi14g00998	14	18,328,471	18,356,529	1509	502
*VvHDA7*	XP_002266492.1	Vitvi15g00473	15	10,328,819	10,349,872	1293	431
*VvHDA8*	XP_002274270.2	Vitvi17g00365	17	4,195,887	4,225,096	2094	698
*VvHDA9*	XP_019082031.1	Vitvi17g00705	17	8,144,795	8,155,931	1941	646
*VvHDA10*	XP_010663108.1	Vitvi17g00894	17	10,614,427	10,630,758	1098	366
**SRT gene family**							
*VvSRT1*	XP_010652928.1	Vitvi07g01918	7	18,710,375	18,723,402	1266	422
*VvSRT2*	XP_010644574.1	Vitvi19g00685	19	8,637,608	8,659,887	1404	468
**HDT gene family**							
*VvHDT1*	XP_002270966.2	Vitvi06g01399	6	19,041,783	19,047,544	1062	354
*VvHDT2*	XP_010654035.1	Vitvi08g01518	8	17,962,761	17,965,626	342	114

### Phylogenetic and exon-intron analysis

**Fig. 1:**
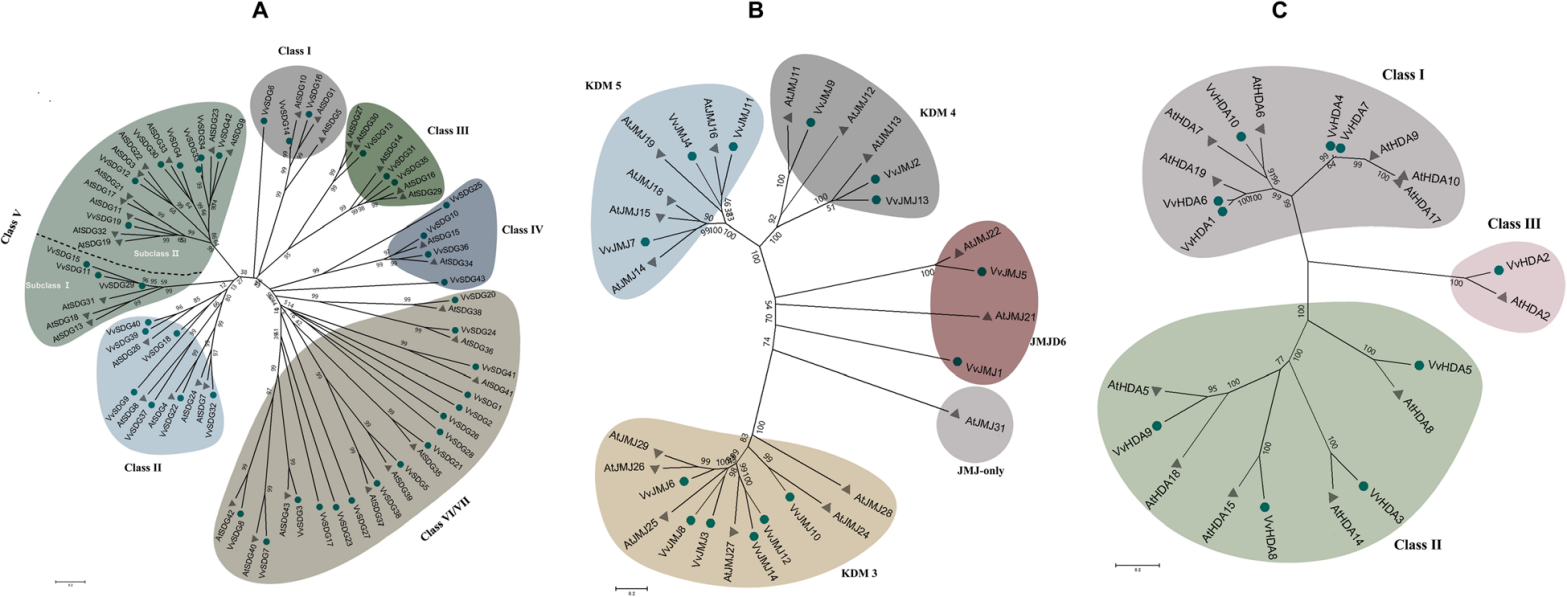
Phylogenetic trees of SDG (**a**), JMJ (**b**), and HAD (**c**) proteins of grapevine and Arabidopsis. Circles and pyramids denote grapevine and Arabidopsis proteins, respectively. Number near the tree branches represent bootstrap values

**Fig. 2:**
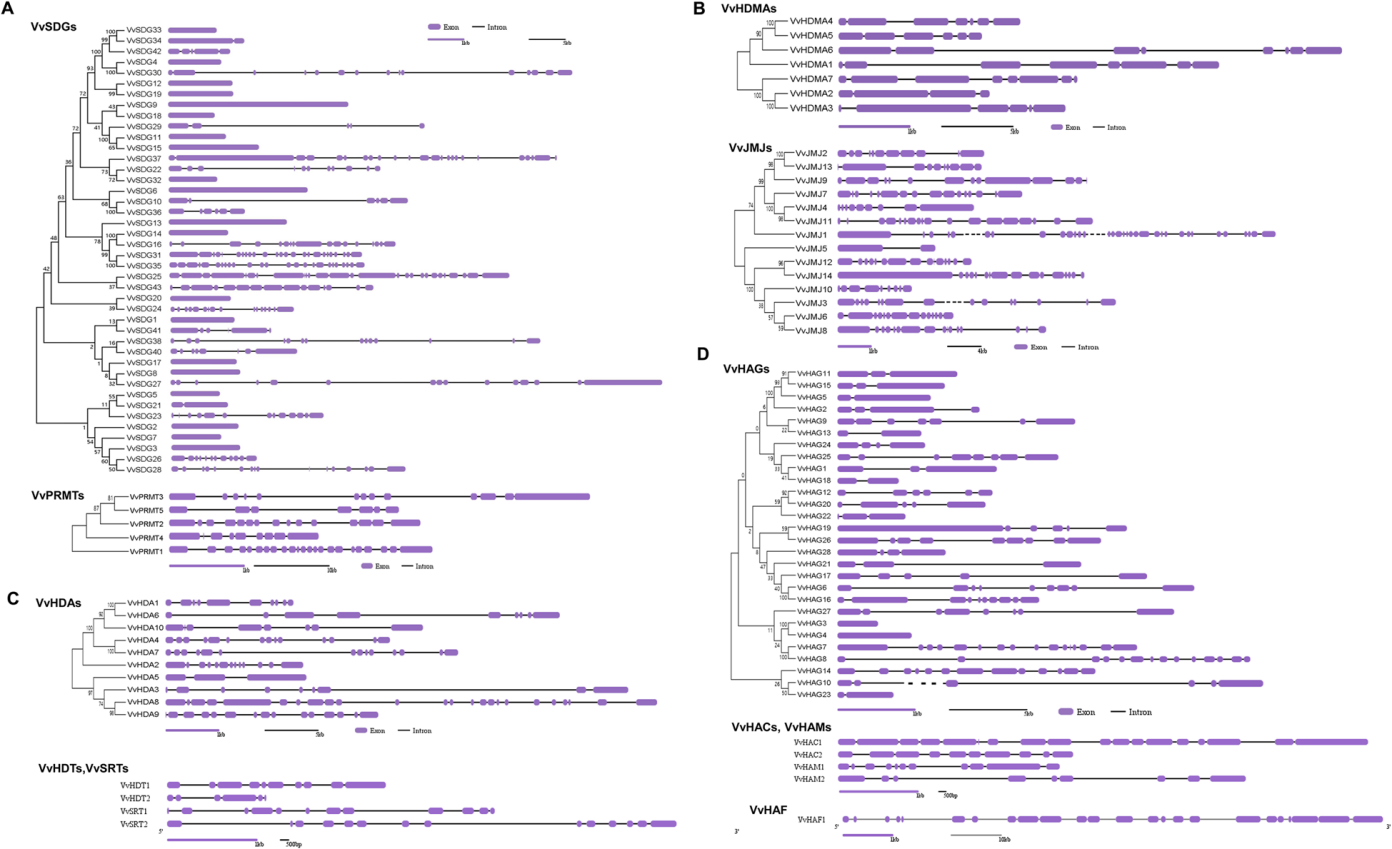
Phylogenetic tree and exon-intron distribution of HMs genes. **a** SDGs and PRMTs; **b** HDMAs and JMJs; **c** HDAs, SRTs, and HDTs; **d** HAGs, HAMs, HACs, and HAFs

To illustrate the phylogenetic history and to classify the HMs gene into different subfamilies, phylogenetic trees were constructed between Arabidopsis and grapevine HMs protein sequences (Fig. 1). Moreover, to deeply study the phylogenetic realtionships unrooted trees were generated among *VvHMS* genes of same group, and intron-exon analysis was performed (Fig. 2). For generation of phylogenetic trees among *VvHMS* genes of same group, we followed the previous trend. Phylogenetic trees were constructed only among members of three well studied subfamilies.

### *VvHMTs*

All 43 SDG genes were divided into seven classes on the basis of phylogenetic tree constructed between Arabidopsis and grape (Fig. 1a). Class V was further subdivided into subclass I and subclass II. Class I was found more conserved with respect to gene numbers, as grape and Arabidopsis contained same; 3 number of genes. Further, different classes contained different number of exons but almost same number of exons with in the same class e.g. class I. However, all five members of PRMTs contained different number of exons, suggesting that PRMTs class is less conserved. Same number of HMTs genes were found in Arabidosis and grapevine. These results suggest that HMTs family is more conserved as compared to other families.

### *VvHDMs*

A phylogenetic tree was constructed between JMJ proteins of grapevine and Arabidopsis. According to the Fig. 1b, JMJ grapevine and Arabidopsis proteins were clustered into 4 and 5 different clades, respectively. Class I (JMJ- domain only), has only one member which is from Arabidopsis. Class II (KDM3), class III (KDM4), and class V (JMJD6) all have equal number of proteins in both species 6, 3, and 2, respectively. However, class IV (KDM5) contained 5 and 3 proteins of Arabidopsis and grapes, respectively. These results suggest that class II, III, and V are more conserved with respect to protein number. Further, exon-intron distribution and size was not same in the same class. However, all duplicated gene pairs have similar exon-intron structure (*VvJMJ2*/*VvJMJ13*, *VvJMJ4*/*VvJMJ11*, and *VvJMJ12*/*VvJMJ14*), with multiple exons and introns, but have difference in size and distribution of exons. Grapevine and Arabidopsis contained 7 and 4 HDMAs proteins, respectively.

### *VvHATs* and *VvHDACs*

In HATs family, HAG subgroup contained the highest (28) number of genes among all groups. In HATs group more difference in number of genes was noticed between grape and Arabidopsis as compared to other groups, suggesting that HATs group have underwent more changes during the process of evolution. Tandemly duplicated gene pairs (*VvHAG3*/*VvHAG4* and *VvHAG7*/*VvHAG8*) showed conserved exon-intron distribution. Moreover, the HAG proteins have high bootstrap values, suggesting high similarity with each other. According to the phylogenetic analysis (Fig. 1c), genes of *VvHDA* were further divided into three classes. Interestingly, class III contained only 2 members, one from each grape and Arabidopsis.

### GO (gene ontology) analysis of HMs

We performed in silico GO analysis of grapevine genes at biological, cellular, and molecular level (Additional file 2: Fig. S1). Prediction of biological process suggested the involvement of HMs genes in regulation of gene expression, cellular component organization, flower development, response to biotic and abiotic stimulus, protein modification processes, and transcription process. Most of the HMs proteins were found in cytoplasm, nucleus, and intracellular organelles. HMs genes might have a role in binding (chromatin, DNA, and protein) transcription and transferase regulation activities at molecular level.

### Chromosomal distribution and expansion pattern of HMs genes in grapevine

**Fig. 3:**
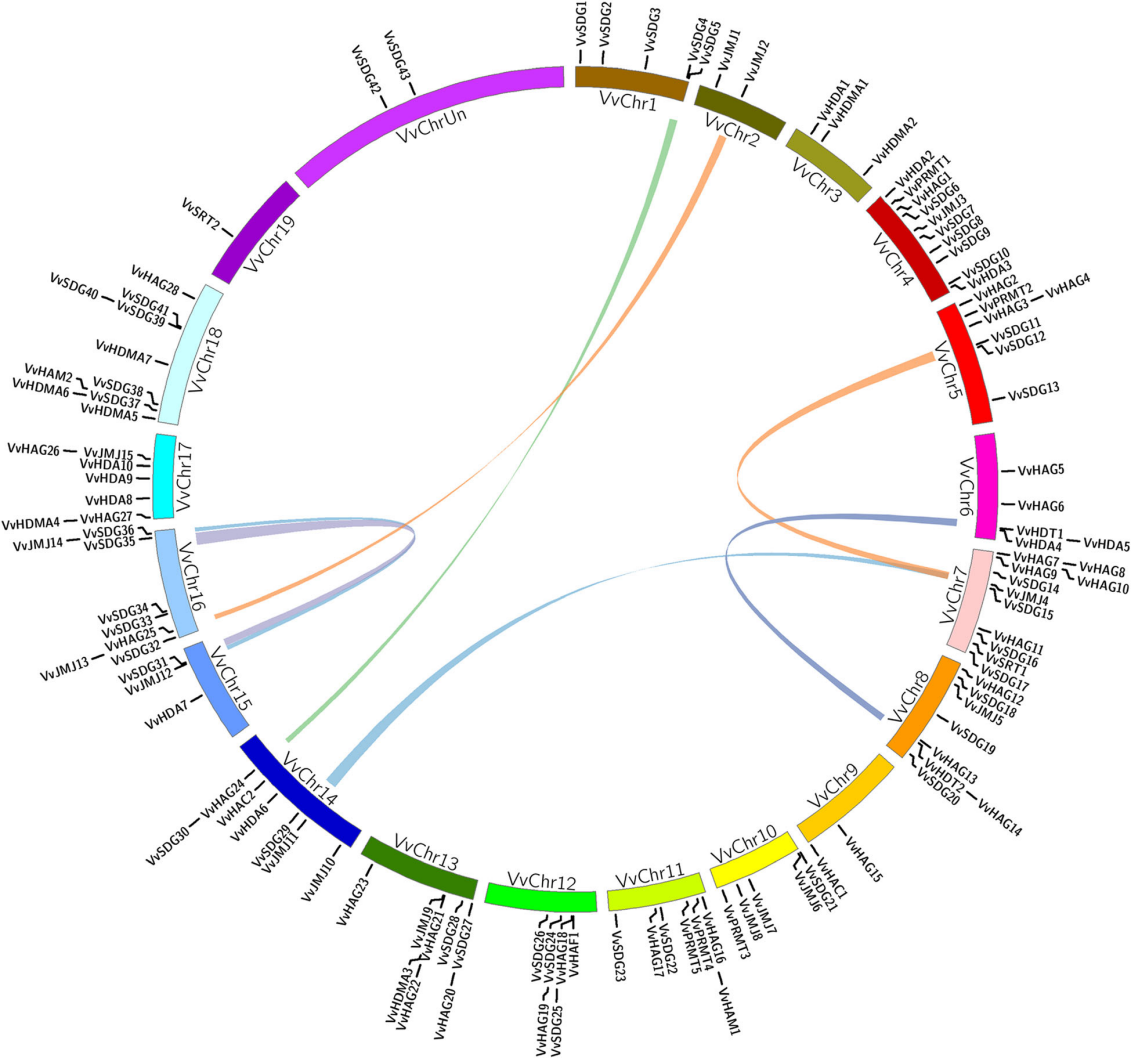
Synteny analysis and chromosomal distribution of grapevine HMs genes. Colored bars connecting two chromosomal regions denote syntenic regions; the corresponding genes on two chromosomes were regarded as segmental duplications. Chr: chromosomes

All 117 HMs genes were unevenly distributed on 20 chromosomes (Fig. 3). Chromosome seven contained the highest number of genes (11) while chromosome 19 contained the lowest only one gene. The 43 *VvSDG* genes were randomly distributed on 14 chromosomes, because chromosomes 2, 3, 6, 9, 17, and 19 has no genes. Four chromosomes 1, 4, 16, and 18 contained equal number of genes; five. These four chromosomes contained almost 50% of genes. For all other gene families, same trend of uneven distribution was noticed (Fig. 3). Tandem duplication was calculated according to the criteria described in materials and methods.

Only three events of tandem duplication (*VvHAG3*/*VvHAG4*, *VvHAG7*/*VvHAG8*, and *VvSDG39*/*VvSDG40*) were observed among six genes on three chromosomes. Two duplication pairs belonged to HAG family, suggesting that this family is less conserved during the evolutionary process. We also calculated the segmental duplication among HMs grapevine genes. Total 15 genes underwent segmental duplication in the form of eight pairs; *VvSDG4*/*VvSDG30*, *VvSDG11*/*VvSDG35*, *VvSDG29*/*VvSDG15*, *VvSDG31*/*VvSDG15*, *VvJMJ2*/*VvJMJ13*, *VvJMJ11*/*VvJMJ4*, *VvJMJ12*/*VvJMJ14*, and *VvHDT1*/*VvHDT2*. Interestingly, *VvSDG15* paired with two genes, *VvSDG29* and *VvSDG31*. These results suggest that both tandem and segmental duplication (21%) events have played a role in the expansion of HMs genes. These results can provide clues for evolutionary history and functional analysis. The ratio between Ka and Ks provides help to describe the evolutionary process [41]. Furthermore, Ka/Ks ratios of all duplicated genes were less than 1, suggesting that all genes have purifying selection pressure (Additional file 3: Table S2).

### Evolutionary relationships between grapes and Arabidopsis

The roles of HMs genes have been well studied in model plants e.g. Arabidopsis and rice. Therefore, to gain insights about the origin and potential functions of HMs genes in grapevine and to explore the evolutionary history, we performed synteny analysis of grapes and Arabidopsis.

**Fig. 4:**
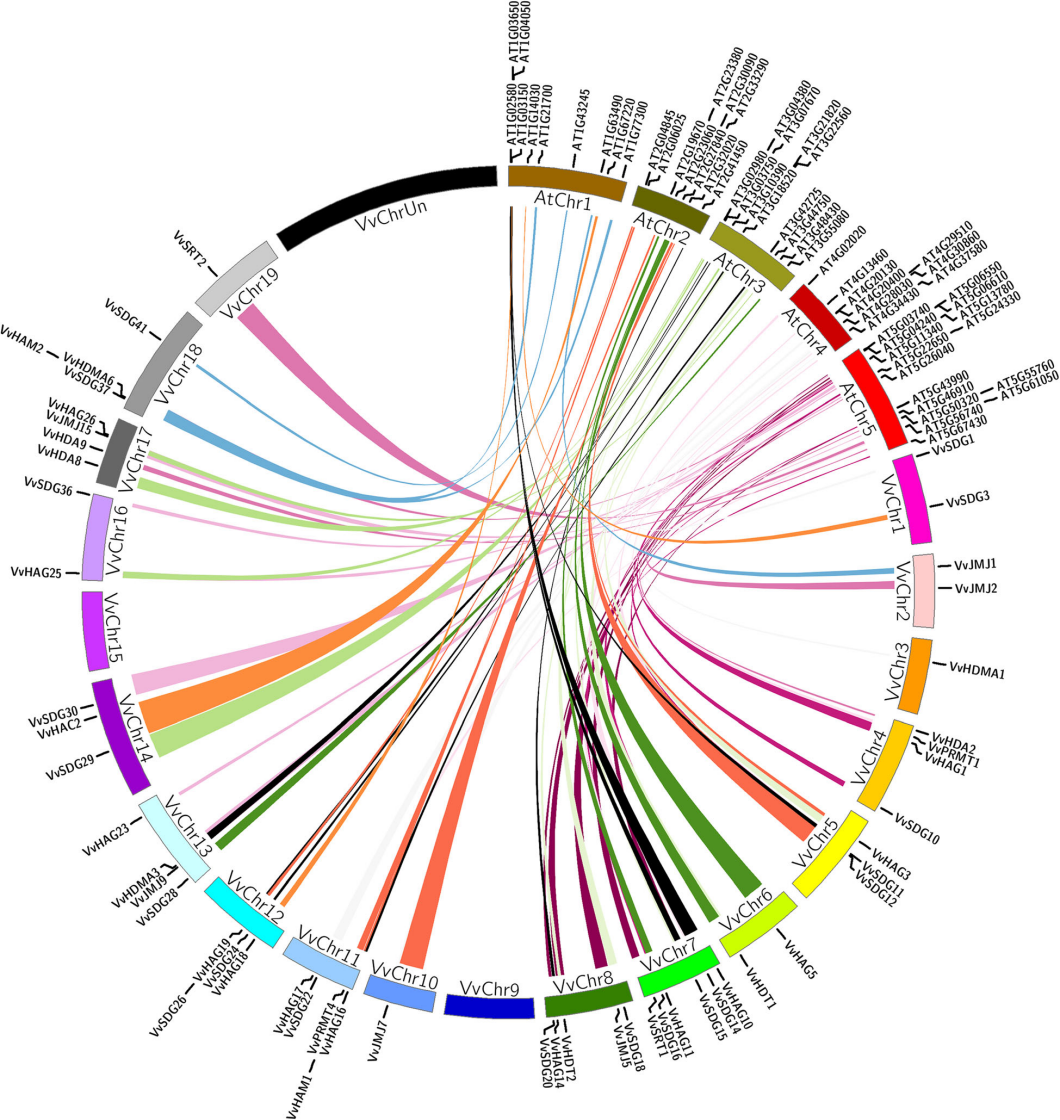
Synteny analysis of HMs genes between Arabidopsis and grapevine. Relative positions were depicted according to the grape and Arabidopsis chromosomes; colored lines represent syntenic regions

As shown in Fig. 4, a total of 69 orthologous pairs were identified between HMs genes of Arabidopsis and grapes comprising of 24, 3, 7, 3, 18, 2, 1, 3, 2, and 6 pairs of SDGs, PRMTs, JMJs, HDMAs, HAGs, HAMs, HACs, HDAs, SRTs, and HDTs, respectively (Additional file 4: Table S3). However, HAFs genes have no syntenic block. There were 24 syntenic blocks of SDG genes having 21 Arabidopsis and 19 grapevine genes. Moreover, there were four pairs of SDG, two pairs of PRMT, one pair of JMJ, four pairs of HAG, and two pairs of HDT, where a single grapevine gene paired with more than one gene of Arabidopsis. There were also five HMs Arabidopsis (3 SDG and 2 HDT) genes, which formed pair with two grapevine genes. All of the pairs contained members from the same subfamily, suggesting that they have a common origin before speciation.

### Transcriptomic analysis of HMs genes during seed development

**Fig. 5:**
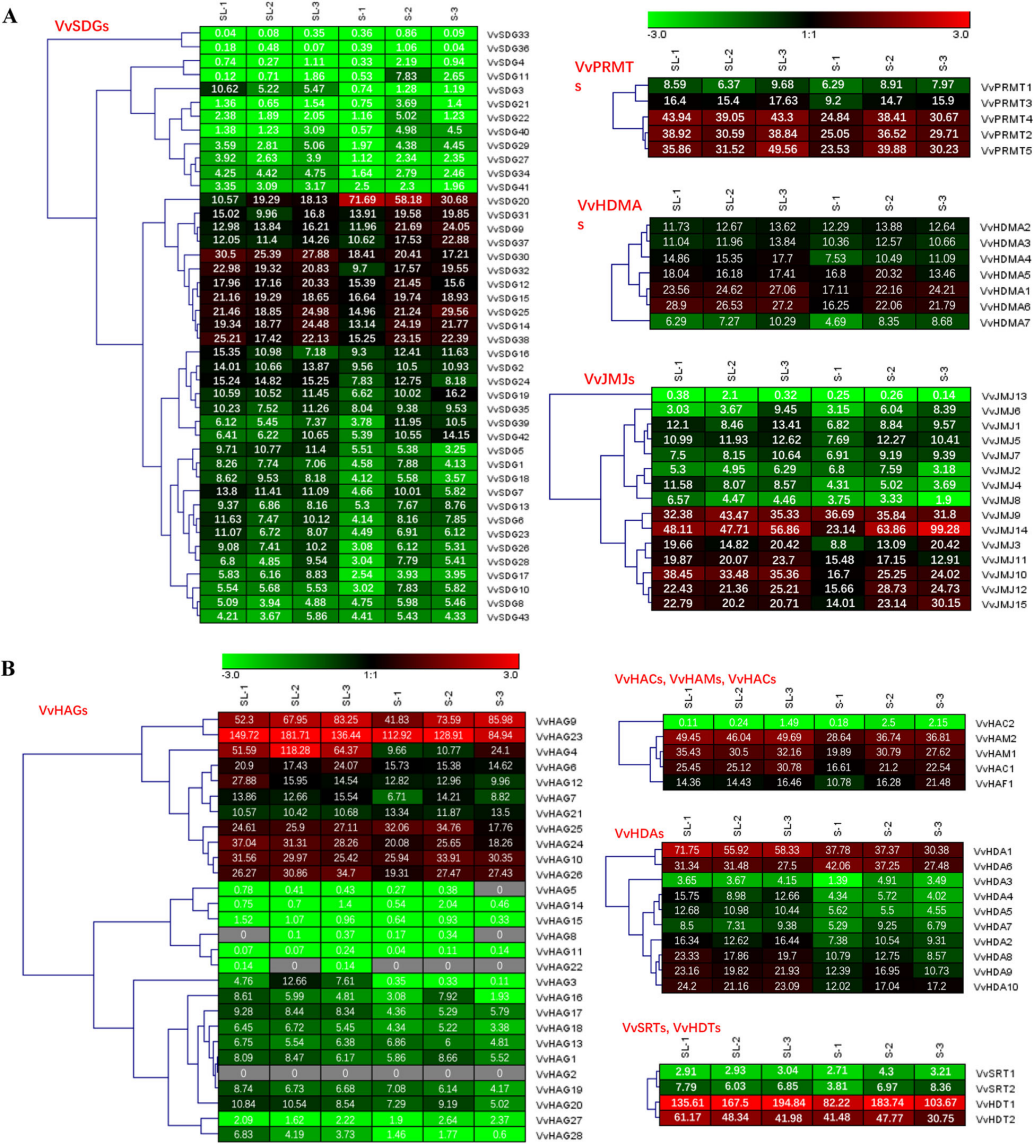
Heat map of *VvHMs* expression in transcriptome analysis during seed development in seeded and seedless progenies. Red and green color scale indicates high and low expression levels, respectively. Grey color indicates barely no gene expression detection. RPKM of *VvHMs* were indicated on the squares. SL1, SL2, and SL3 denotes Seedless progeny at seed developmental stage 1, 2, and 3, respectively. S1, S2, and S3 denotes Seeded progeny at seed developmental stage 1, 2, and 3, respectively

To get some clues about the potential roles of HMs grapevine genes in seed development, transcriptomic analysis of HMs grapevine genes was performed (Fig. 5 and Additional file 5: Table S4). The samples were taken at three different stages of seed development from seeded and seedless progenies. Most of the HMs genes were highly expressed during successive stages of seed formation in both seeded and seedless progenies. However, most of the HDA family members were expressed relatively very low. There were some exceptions as well, for example, *VvJMJ14* showed up-regulation during the later stages of seed development in seeded progenies as compared to seedless. In contrast, *VvSDG30*, *VvHDA1*, and *VvHAG23* were highly expressed during all stages of seed development in seedless cultivars, suggesting their potential role in ovule abortion. Therefore, we speculate that HMs genes have potential roles in seed development and ovule abortion. We observed that some of the grapevine HMs gene pairs generated from duplication events showed similar expression patterns in gene pairs. For example, most of the duplicated gene pairs of JMJ family were conserved with respect to exon-intron distribution and expression pattern. However, there were some exceptions as well, *VvSDG4*/*VvSDG30* and *VvSDG15*-*VvSDG29*/*VvSDG31* (Segmentally), i.e. *VvSDG4* and *VvSDG30* has significantly different expression during progressive stages of seed development. Further with regard to duplicated genes, JMJ subfamily is more conserved as compared to SDG subfamily.

### Expression pattern of HMs genes during different stages of seed development in different grape cultivars

**Fig. 6:**
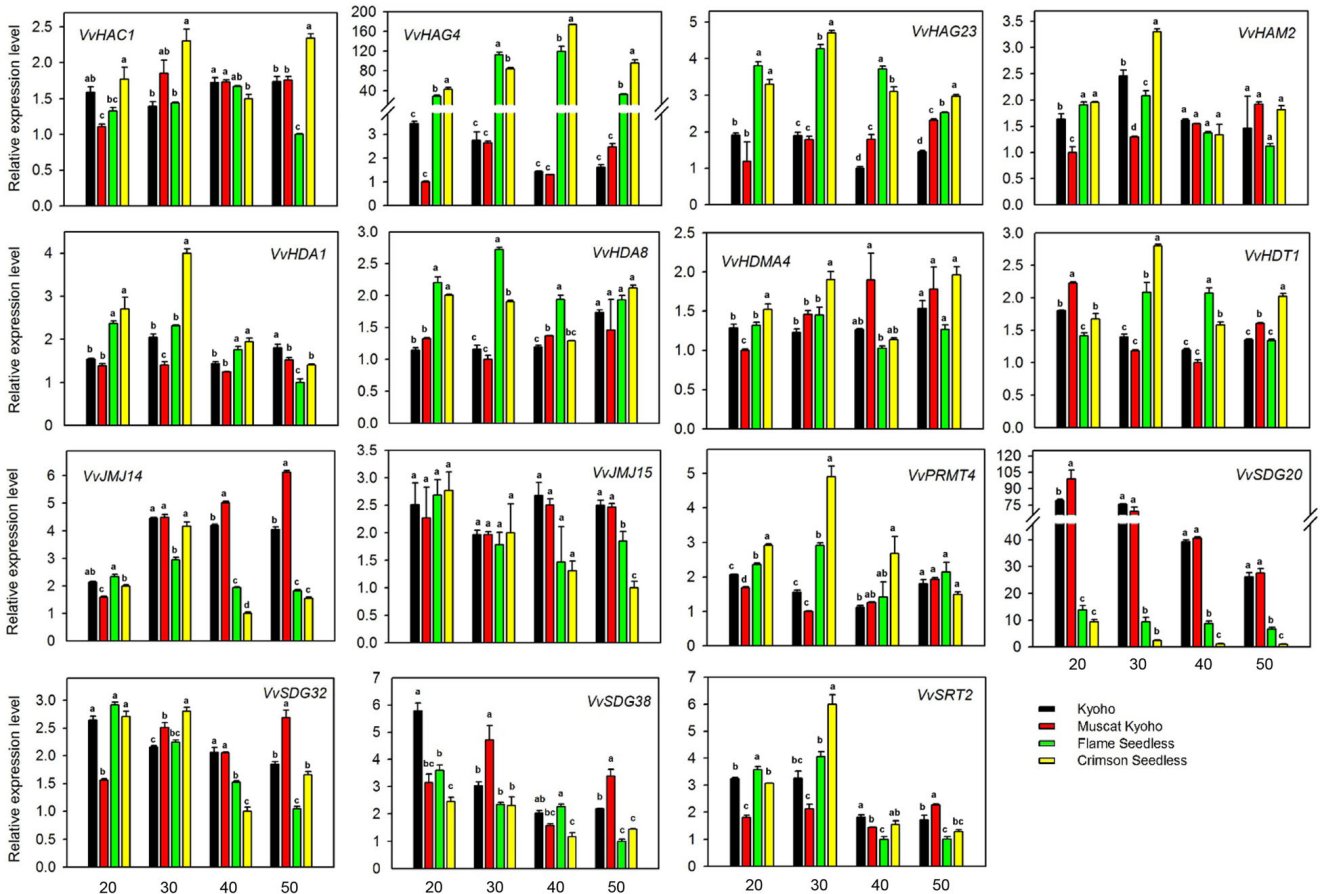
Real-time PCR analysis of *VvHMs* genes during seed development in four cultivars. Different colors denote different cultivars and numbers on X-axis represent days after flowering (DAF). Different letters indicate statistically significant differences (ANOVA with a Tukey post-hoc analysis, 5% level). The mean ± S.D. of three biological replicates are presented

To verify the reliability of transcriptome data, and to explore whether the expression pattern of VvHMs genes is widespread in different grape varieties, we selected 15 HMs genes and analyzed the expression pattern in different seeded (‘Kyoho’ and ‘Muscat kyoho’) and seedless (‘Flame seedless’ and ‘Crimson seedless’) grape cultivars during successive stages of seed development. The genes were selected according to the results of transcriptome data of seed development (Fig. 5). These genes were comprised of representatives of all subfamilies including differently (*VvHAC1, VvHAG23*, *VvHAM2, VvHDA1, VvHDA8, VvHDT1, VvJMJ14, VvJMJ15, VvSDG20, VvSDG32*, and *VvSDG38*) and ubiquitously (*VvHAG4, VvHDMA4, VvPRMT4*, and *VvSRT2*) expressed genes during different stages of seed development in seeded and seedless cultivars. As shown in Fig. 6, *VvJMJ14, VvJMJ15,* and *VvSDG38*, were significantly highly expressed during later stages of seed development in seeded cultivars. *VvSDG20* showed significantly higher expression during all stages of seed development in seeded cultivars compared to seedless ones. Further, the change ratio between seeded and seedless cultivars was relatively higher in *VvSDG20* as compared to other differentially expressed genes. Based on the results, we speculate that these genes might have a role in seed development. In contrast to these results, some genes were signficantly highly expressed in some stages of seed formation in seeedless cultivars, for example, *VvHAG23*, *VvHDA8,* and *VvPRMT4*. Moreover, *VvHAG4* showed remarkable difference in the expression pattern during all stages of seed development in seedless cultivars than seeded ones. These results suggest that these genes might have a role in ovule abortion. In general, the results of qRT-PCR are consistent with the results of transcriptome.

### Expression profiling of HMs genes in response to *E*. *necator* inoculation and hormone treatments

**Fig. 7:**
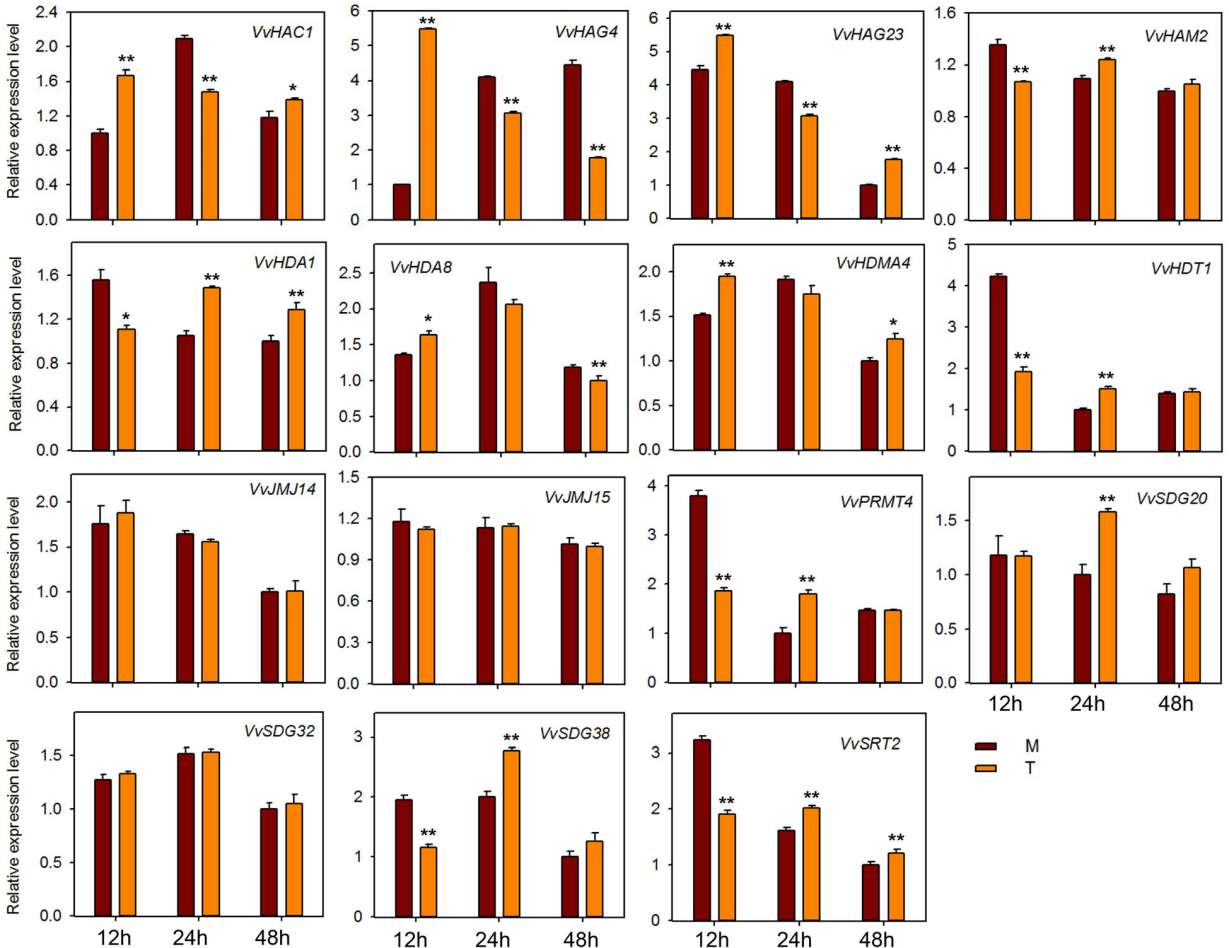
Real-time PCR expression analysis of *VvHMs* genes following *Erysiphe necator* inoculation at 12, 24, and 48 h post inoculation. M and T denotes mock and treatment, respectively. Asterisks indicate the corresponding statistically significance (ANOVA with a Tukey post-hoc analysis, *P < 0.05, **P < 0.01). The mean ± S.D. of three biological replicates are presented

**Fig. 8:**
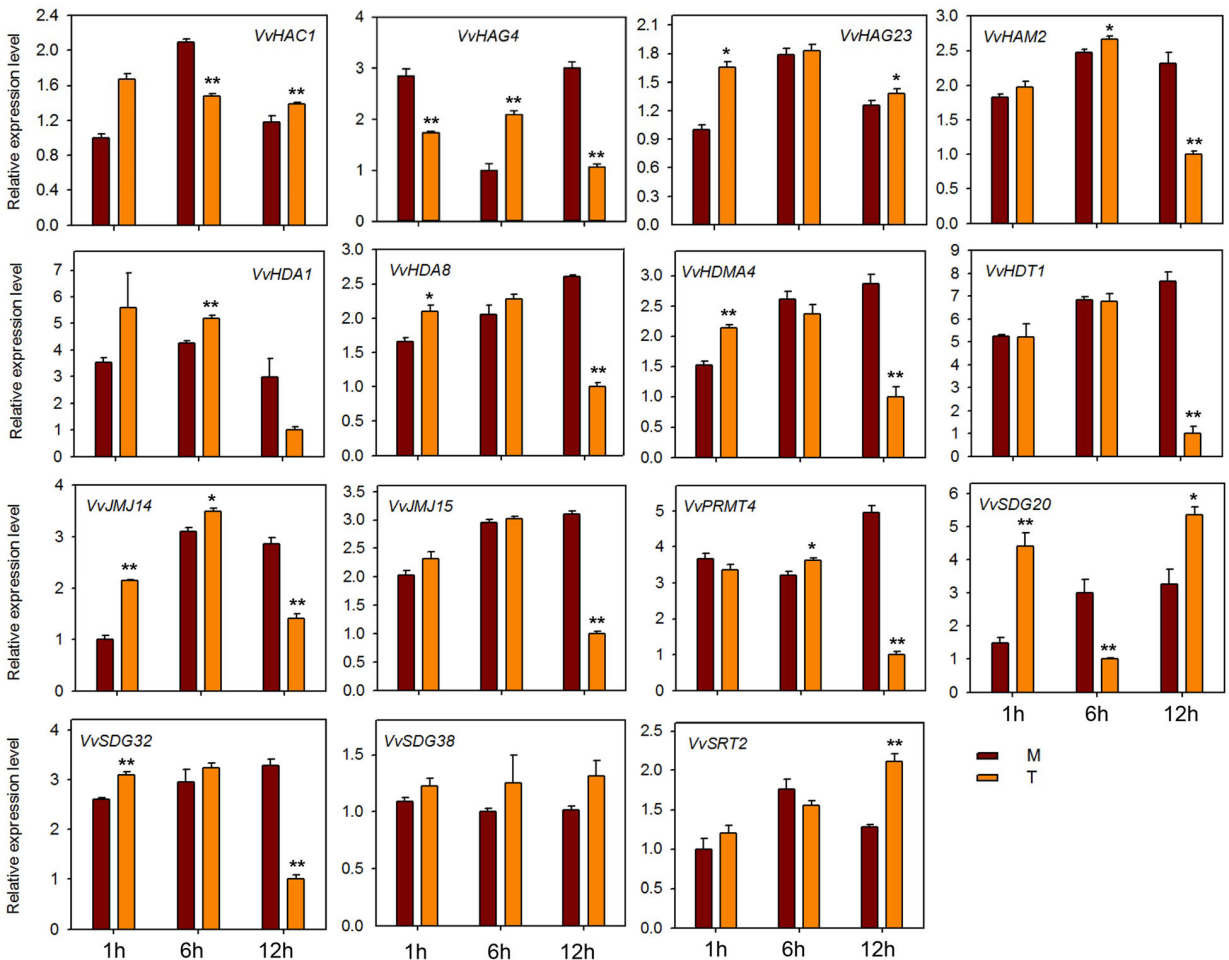
Real-time PCR expression analysis of *VvHMs* genes following ABA treatment. Leaves were collected at 1, 6, and 12 h after treatment. M and T denotes mock and treatment, respectively. Asterisks indicate the corresponding statistically significance (ANOVA with a Tukey post-hoc analysis, *P < 0.05, **P < 0.01). The mean ± S.D. of three biological replicates are presented

**Fig. 9:**
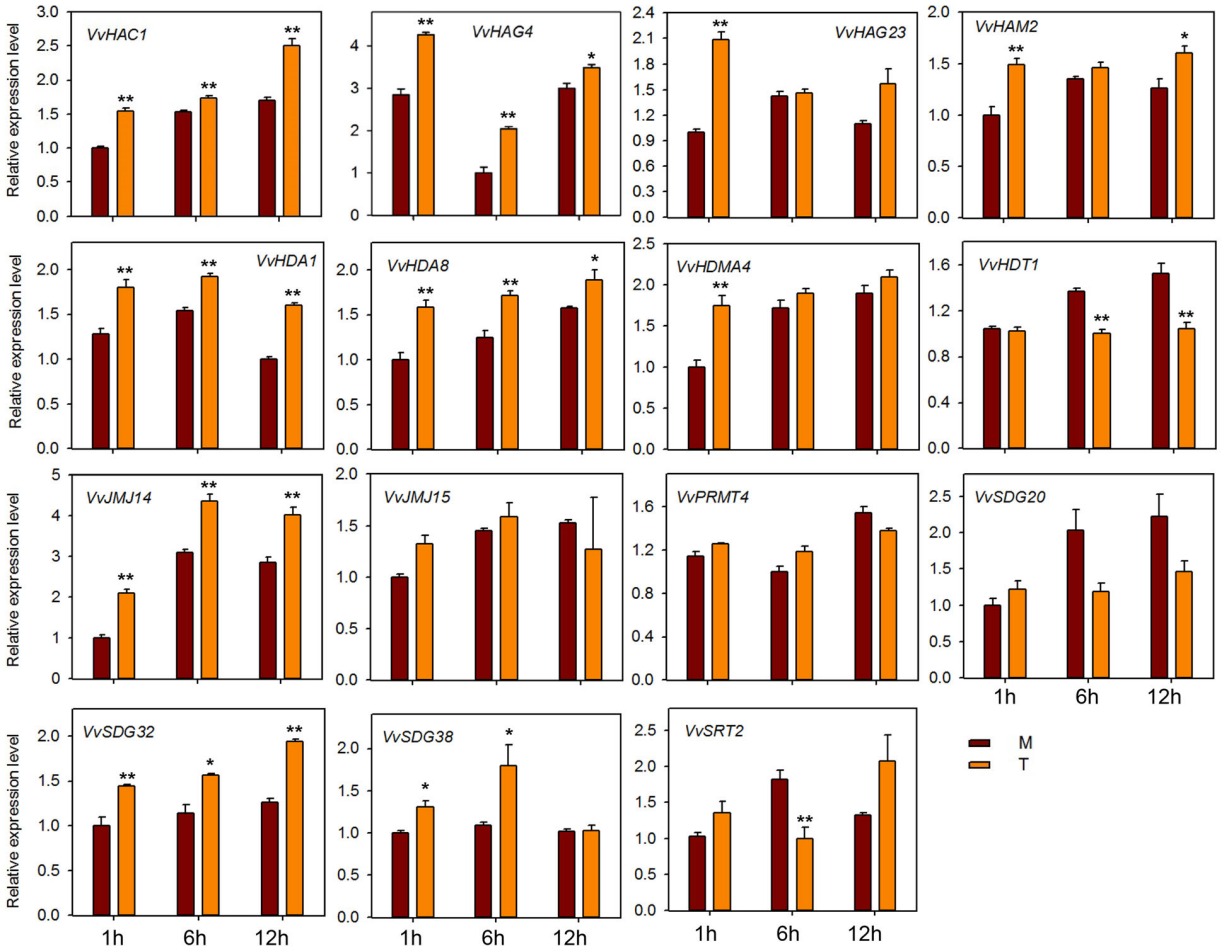
Real-time PCR expression analysis of *VvHMs* genes following JA treatment. Leaves were collected at 1, 6, and 12 h after treatment. M and T denotes mock and treatment, respectively. Asterisks indicate the corresponding statistically significance (ANOVA with a Tukey post-hoc analysis, *P < 0.05, **P < 0.01). The mean ± S.D. of three biological replicates are presented

**Fig. 10:**
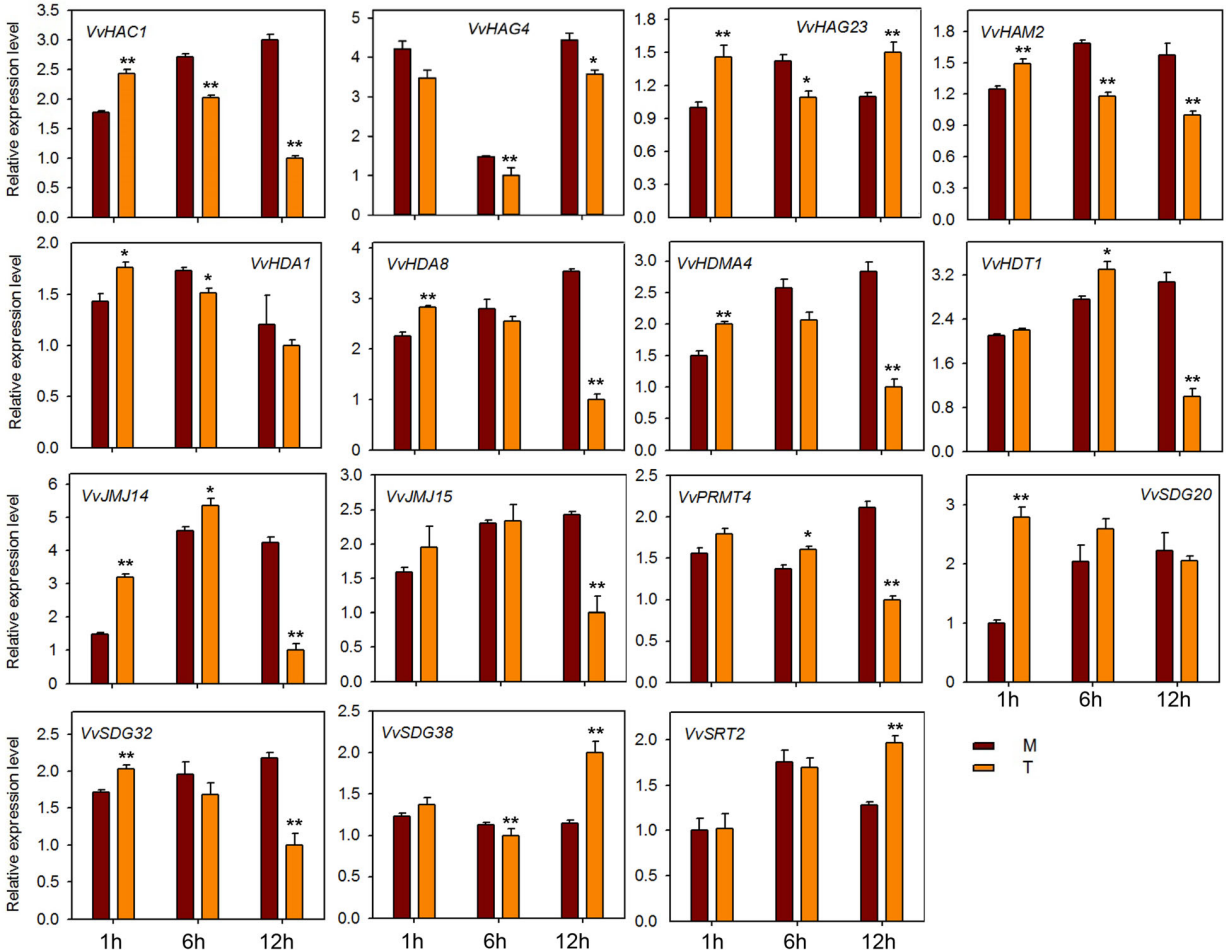
Real-time PCR expression analysis of *VvHMs* genes following SA treatment. Leaves were collected at 1, 6, and 12 h after treatment. M and T denotes mock and treatment, respectively. Asterisks indicate the corresponding statistically significance (ANOVA with a Tukey post-hoc analysis, *P < 0.05, **P < 0.01). The mean ± S.D. of three biological replicates are presented

**Fig. 11:**
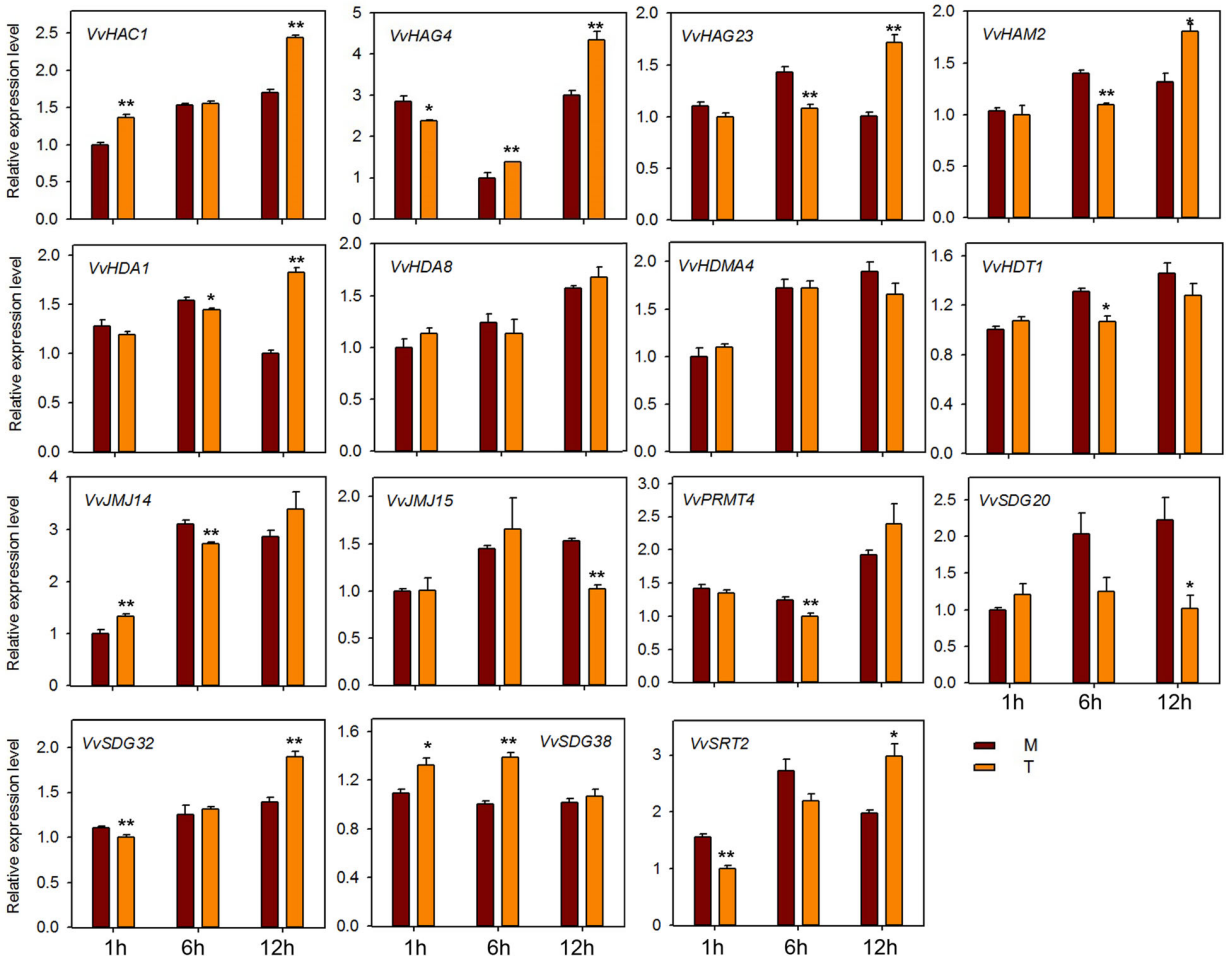
Real-time PCR expression analysis of *VvHMs* genes following Ethylene treatment. Leaves were collected at 1, 6, and 12 h after treatment. M and T denotes mock and treatment, respectively. Asterisks indicate the corresponding statistically significance (ANOVA with a Tukey post-hoc analysis, *P < 0.05, **P < 0.01). The mean ± S.D. of three biological replicates are presented

To investigate the potential role of HMs genes against powdery mildew infection, we performed qRT-PCR analysis of 15 HMs genes (same genes as for seed study) after infecting ‘Shang-24’ with *Erysiphe necator*. At 12 hpi the expression of three genes (*VvHAC1*, *VvHAG4,* and *VvHAG23*) was significantly up-regulated but subsequently down-regulated at 24 hpi (Fig. 7). However, the expression of *VvHAM2, VvHDA1*, *VvPRMT4*, *VvHDT1*, *VvSDG38*, and *VvSRT2* was down-regulated at 12 hpi, and up-regulated at 24 hpi. Moreover, some of the genes (*VvJMJ14*, *VvJMJ15*, and *VvSDG32*) showed no significant difference in expression between treated and control plants. These results suggest that some of the HMs genes are responsive to powdery mildew and might have a role in resistance mechanism. We also checked the responses of 15 HMs grapevine genes to ABA, ethylene, JA, and SA applications. In plants treated with ABA, most of the genes were down regulated at 12 hpi (Fig. 8). But *VvHAC1*, *VvHAG23*, *SDG20*, and *VvSRT2* genes were significantly up-regulated at 12 hpi. *VvHAG4*, *VvJMJ14*, and *VvSDG20* showed significantly differential expression at all-time points. However, *VvSDG38* gene showed same expression in control and treated plants at all-time points, suggesting that, except this all other genes have link with ABA regulation in grapevine. For JA treated plants, *VvHAC1, VvHAG4*, *VvHDA1*, *VvHDA8*, *VvJM14*, and *VvSDG32* showed significant up-regulation at all-time points. However, *VvHAG23* and *VvHDMA4* showed significant up-regulation only at 1 hpi. Further, *VvHDT1* was significantly down-regulated at 6 and 12 hpi after JA treatment (Fig. 9). In SA treated plants, *VvHAC1*, *VvHAG23*, *VvHAM2*, *VvHDA1*, *VvHDA8*, *VvHDMA4*, *VvJMJ14*, *VvSDG20*, and *VvSDG32* gene were significantly up-regulated at 1 hpi and most of these genes were down-regulated at 12 hpi (Fig. 10). Furthermore, seven genes (*VvHAC1*, *VvHAG4*, *VvHAG23*, *VvHAM2*, *VvHDA1*, *VvSDG32*, and *VvSRT2*) showed significantly higher expression at 12 hpi in ethylene treated plants as compared to control (Fig. 11). Moreover, the expression of *VvHAG23*, *VvHAM2*, and *VvHDA1* was similar with each other at all-time points i.e. same expression at 1 hpi, down-regulation at 6 hpi and subsequent up-regulation at 12 hpi. However, *VvJMJ15* and *VvSDG20* showed significant down-regulation at 12 hpi. These results suggest that most of the HMs genes have role in defense mechanism of grapevine in response to *E. necator* inoculation and have involvement in hormone pathways.

## Discussion

HMs gene families have been well studied in model plants and affects many processes during plant growth and development including root growth, flower induction, floral organogenesis, embryo development, and plant responses to biotic and abiotic stresses [42–44]. In recent years HMs genes have been identified in fruit crops including citrus, apple, and strawberry. These studies have reported the putative functions of these genes in flower induction, fruit development, response to blue mold infection, cold, and heat stress [30–32]. However, a limited information is available about the HMs gene numbers and functions in grapevine. In this study, we have described the detailed bioinformatics and expression analysis of HMs genes against *E. necator* inoculation and different hormonal treatments. We identified 117 *VvHMs* genes and divided them into 11 subfamilies on the basis of phylogenetic relationships with Arabidopsis and conserved domains. All the genes were named according to their family and position on the chromosomes [30, 31]. Previously, in grapevine Aquea et al. [45, 46] has reported 7, 13, and 33 genes in HAT, HDAC, and SDG subfamilies, respectively. However, we identified more number of genes as compared to previous studies, the difference in number of genes is due to advancements in gene searching websites and different searching methods. Among all families of VvHMs, the VvSDG family was more conserved and VvHAG was less conserved. Our results are also supported by exon-intron analysis (Fig. 2). Moreover, SDG family is also conserved with respect to gene number in different species i.e. grapevine, Arabidopsis, citrus, and tomato have 43, 41, 40, and 43 SDG genes, respectively [31]. According to predicted protein length HDA subfamily members are more conserved as compared to other subfamilies (Table 1). Further, our findings are concordant with previous findings in other crops [30–32].

Gene duplication has played a vital role in the expansion of gene families [47]. According to studies, segmental and tandem duplication both have significant contributions in the expansion of grapevine gene families [48]. We observed the previous trend with respect to genes distribution i.e. highly variable distribution of genes on chromosomes. The highest number of genes were present on chromosome seven. This suggests that most of the duplication events were occurred on this chromosome. These observations are supported by results as both type of duplication pairs (tandem and segmental) were found on chromosome seven, suggesting that this chromosome has played potential role in the expansion of HMs gene families. Moreover, more duplication events might be the reason of clustering of genes on chromosome but we have no evidences to support this hypothesis [49]. According to results, 21% of the HMs genes are the results of duplication events. We observed more segmental duplication (8 pairs) events as compared to tandem duplication (3 pairs) events. In case of duplication events we observed the previous trend as the segmental duplication has occurred more commonly than tandem duplication during the evolutionary process in plants [47]. Further, pairing of *VvSDG15* with two genes (*VvSDG29* and *VvSDG31*), suggests that it might have diverse roles in plant growth and development. Moreover, more tandem duplication events in VvHAG gene family further justify our results of gene number and exon-intron analysis that HAG gene family is less conserved. Generally, duplicated gene pairs showed conservation with regard to exon-intron distribution and expression pattern. However, there were some differences as well, *VvSDG4*/*VvSDG30* and *VvSDG15*-*VvSDG29*/*VvSDG31* (Segmentally), i.e. *VvSDG15* showed ubiquitous expression during all stages of seed development in both seeded and seedless progenies, while expression of *VvSDG29* was low in seeds of both seeded and seedless progenies (Fig. 5). The duplicated gene pairs of JMJ subfamily were found more conserved in exon-intron distribution and expression pattern. The ratio between Ka and Ks provides help to describe the evolutionary process [42]. According to results all duplicated genes have negative mode of selection. Previously, similar results about HMs genes have been reported in other fruit crops i.e. apple, citrus, and strawberry [30–32].

Synteny analysis is a very important bioinformatic tool as synteny blocks can provide inklings about gene evolution and function [50]. We found 68 synteny blocks (Segmental duplication pairs) between grapes and Arabidopsis HMs genes. Presence of more segmental duplication pairs between grapevine and Arabidopsis HMs genes, suggest that they might have some common origin before speciation. However, it is difficult to explain evolutionary history only on the basis of syntenic relationships. Based on the potential roles of orthologous genes of grapevine in other crops we can predict functions of HMs genes in grapes.

Previously, some transcription factors and gene families have been reported for their roles in grapevine seed development, such as MADS-box, B3, and GASA gene family [33, 34, 41]. In Arabidopsis HMs genes, especially SDG family has been well studied for their potential roles in vegetative and reproductive growth of plant. Expression analysis of genes can provide clues about gene function. Based on presumed roles of HMs genes in other crops, we performed expression profiling of selected HMs grapevine genes at different stages of seed development, against infection of *Erysiphe necator*, and after application of multiple plant hormones. The results were obtained as anticipated, for example *VvSDG20* showed different expression in seeds of seeded and seedless cultivars. According to previous study, *VvSDG12*, *VvSDG14*, *VvSDG16*, *VvSDG25*, and *VvSDG37* (Previously known as SDG6934, SDG66905, SDG66903, SDG66932, and SDG66911) showed high expression in flowers and fruits of grapevine [45]. In our study, these genes showed ubiquitous expression in both seeded and seedless cultivars. Therefore, we speculate that these genes might have a general role in the reproductive process. Plant hormones, such as ABA, ethylene, JA, and SA, play important roles in regulating developmental processes and signaling networks involved in plant responses to biotic and abiotic stresses. In our study, *VvPRMT4* was up-regulated against *Erysiphe necator* and after SA treatments. Further, the expression of *VvSDG32* was also affected by JA and SA applications. Previously, the involvement of SDG genes in embryo development, fruit development, and response to biotic and abiotic stimulus in citrus [30], strawberry [32], and tomato [51] have been reported. In case of HDMs genes (*VvJMJ14*, *VvJMJ15*, and *VvHDMA4*) similar results like HMTs genes were noticed. Therefore, we speculated that HMTs and HDMs genes might have potential roles in seed development and disease resistance via affecting hormonal signaling pathways. In general, our study provides some clues about gene functions based on bioinformatic and expression analysis. A future study is needed to get exact information about gene functions.

*VvHAC1* gene showed differential expression in response to powdery infection, ABA, JA, SA, and ethylene application in treated and control plants. Li et al. [52] reported the involvement of *AtHAC1* (orthologous of *VvHAC1*) in ethylene signaling pathway. Further, mutants of *AtHAC1* showed resistance to high concentrations of glucose during early stages of root development. *VvHAM2* was up-regulated against JA and SA exogenous application and showed significant down-regulation to exogenous application of ethylene. In Arabidopsis, *HAM1/2* has been reported for their involvement in male and female gametogenesis [16]. These results suggest that HATs genes play vital role in plant development via regulating gene expression. HDACs genes also play potential roles in the regulation of gene expression and chromatin structure [46]. For example, *AtHDA19* played significant roles in plant defense via regulating multiple hormonal signaling pathways and networking with *WRKY38* and *WRKY62*. In our studies, *VvHDA1* (orthologous of *AtHDA19*) showed significant change in expression against disease attack and hormonal applications, suggesting its involvement in multiple biological processes. Moreover, *VvSRT2* showed significant down-regulation against powdery mildew attack and ethylene application. Similar type of results were obtained for SRT genes functions in Arabidopsis and rice [53, 54]. Finally, our study provides inklings about functions of HMs genes in grapevine.

## Conclusion

In this study, we identified 117 *HMs* genes in grapes. We divided the genes into four subgroups and subsequently divided these groups into 11 subfamilies. Further, we analyzed the genes in terms of chromosomal locations, exon-intron distribution, and evolutionary history. According to evolutionary history, the segmental and tandem duplication both have played role in the expansion of *HMs* genes in grapevine. According to the results, *VvHMs* genes might have role in seed development, disease resistance, and multiple hormonal signaling pathways. Our study is useful for functional characterization of *VvHMs* genes.

## Supplementary information

**Additional file 1: Table S1.** The primer sequences used for qRT-PCR amplification.

**Additional file 2: Figure S1.** Gene ontology analysis of *VvHMs*.

**Additional file 3: Table S2.** Synteny blocks of HM genes within grape genome.

**Additional file 4: Table S3.** Synteny blocks of HM genes between grape and Arabidopsis genome.

**Additional file 5: Table S4.** Detailed information of DEGs identified in transcriptome analysis.

## Data Availability

The datasets supporting the conclusions of this article are included within the article and its additional files.

## Abbreviations

ABA: Abscisic acid
BLAST: Basic Local Alignment Search Tool
CBP: (cAMP responsive element-binding protein)-binding protein
CLF: CURLY LEAF
DAF: Days after flowering
Eth: Ethylene
GNAT: General control non repressible 5-related N-terminal acetyltransferase
GO: Gene ontology
HATs: Histone acetylases
HDACs: Histone deacetylases
HDMs: Histone methylases
HMs: Histone modifications
HMTs: Histone methyltransferases
HMM: Hidden Markov Model
JMJ: JmjC domain-containing proteins
Ka: Non-synonymous substitution rate
Ks: Synonymous substitution rate
MEA: MEDEA
MeJA: Methyl jasmonate
NJ: Neighbor-joining
PRMTs: Protein arginine methyltransferases
RPD3: Reduced Potassium Dependence
SA: Salicylic acid
SDG: Set domain group
SIR2: Silent information regulator 2
TAF_II_250: TATA-binding protein-associated factor

## References

[1] Yuan L, Liu X, Luo M, Yang S, Wu K (2013). Involvement of histone modifications in plant abiotic stress responses. J Integr Plant Biol.

[2] Cedar H, Bergman Y (2009). Linking DNA methylation and histone modification: patterns and paradigms. Nat Rev Genet.

[3] Liu X, Yang S, Yu CW, Chen CY, Wu K (2016). Histone acetylation and plant development. Enzymes.

[4] Liu C, Lu F, Cui X, Cao X (2010). Histone methylation in higher plants. Annu Rev Plant Biol.

[5] Dong G, Ma D-P, Li J (2008). The histone methyltransferase SDG8 regulates shoot branching in *Arabidopsis*. Biochem Biophys Res Commun.

[6] Cazzonelli CI, Cuttriss AJ, Cossetto SB, Pye W, Crisp P, Whelan J, Finnegan EJ, Turnbull C, Pogson BJ (2009). Regulation of carotenoid composition and shoot branching in *Arabidopsis* by a chromatin modifying histone methyltransferase, SDG8. Plant Cell.

[7] Cazzonelli CI, Nisar N, Roberts AC, Murray KD, Borevitz JO, Pogson BJ (2014). A chromatin modifying enzyme, SDG8, is involved in morphological, gene expression, and epigenetic responses to mechanical stimulation. Front Plant Sci.

[8] Pandey R, Muller A, Napoli CA, Selinger DA, Pikaard CS, Richards EJ, Bender J, Mount DW, Jorgensen RA (2002). Analysis of histone acetyltransferase and histone deacetylase families of *Arabidopsis thaliana* suggests functional diversification of chromatin modification among multicellular eukaryotes. Nucleic Acids Res.

[9] Feng Q, Wang HB, Ng HH, Erdjument-Bromage H, Tempst P, Struhl K, Zhang Y (2002). Methylation of H3-lysine 79 is mediated by a new family of HMTases without a SET domain. Curr Biol.

[10] Goodrich J, Puangsomlee P, Martin M, Long D, Meyerowitz EM, Coupland G (1997). A polycomb-group gene regulates homeotic gene expression in *Arabidopsis*. Nature.

[11] Grossniklaus U, Vielle-Calzada JP, Hoeppner MA, Gagliano WB (1998). Maternal control of embryogenesis by *MEDEA*, a *Polycomb* group gene in *Arabidopsis*. Science.

[12] Avramova Z (2015). Transcriptional ‘memory’ of a stress: transient chromatin and memory (epigenetic) marks at stress-response genes. Plant J.

[13] Thorstensen T, Grini PE, Aalen RB (2011). SET domain proteins in plant development. BBA Gene Regul Mech.

[14] Zhao Z, Yu Y, Meyer D, Wu C, Shen W-H (2005). Prevention of early flowering by expression of *FLOWERING LOCUS C* requires methylation of histone H3 K36. Nat Cell Biol.

[15] Pei Y, Niu L, Lu F, Liu C, Zhai J, Kong X, Cao X (2007). Mutations in the type II protein arginine methyltransferase AtPRMT5 result in pleiotropic developmental defects in Arabidopsis. Plant Physiol.

[16] Latrasse D, Benhamed M, Henry Y, Domenichini S, Kim W, Zhou D-X, Delarue M (2008). The MYST histone acetyltransferases are essential for gametophyte development in Arabidopsis. BMC Plant Biol.

[17] Wu KQ, Tian LN, Malik K, Brown D, Miki B (2000). Functional analysis of HD2 histone deacetylase homologues in *Arabidopsis thaliana*. Plant J.

[18] Cigliano RA, Sanseverino W, Cremona G, Ercolano MR, Conicella C, Consiglio FM (2013). Genome-wide analysis of histone modifiers in tomato: gaining an insight into their developmental roles. BMC Genomics.

[19] Yano R, Takebayashi Y, Nambara E, Kamiya Y, Seo M (2013). Combining association mapping and transcriptomics identify *HD2B* histone deacetylase as a genetic factor associated with seed dormancy in *Arabidopsis thaliana*. Plant J.

[20] Song XJ, Kuroha T, Ayano M, Furuta T, Nagai K, Komeda N, Segami S, Miura K, Ogawa D, Kamura T (2015). Rare allele of a previously unidentified histone H4 acetyltransferase enhances grain weight, yield, and plant biomass in rice. P Natl Acad Sci USA.

[21] Liu XC, Chen CY, Wang KC, Luo M, Tai R, Yuan LY, Zhao ML, Yang SG, Tian G, Cui YH (2013). PHYTOCHROME INTERACTING FACTOR3 associates with the histone deacetylase HDA15 in repression of chlorophyll biosynthesis and photosynthesis in etiolated *Arabidopsis* seedlings. Plant Cell.

[22] Benhamed M, Bertrand C, Servet C, Zhou D (2006). *Arabidopsis GCN5, HD1,* and *TAF1/HAF2* interact to regulate histone acetylation required for light-responsive gene expression. Plant Cell.

[23] Liu X, Luo M, Zhang W, Zhao JH, Zhang JX, Wu KQ, Tian LN, Duan J (2012). Histone acetyltransferases in rice (*Oryza sativa* L.): phylogenetic analysis, subcellular localization and expression. BMC Plant Biol.

[24] Long JA, Ohno C, Smith ZR, Meyerowitz EM (2006). TOPLESS regulates apical embryonic fate in *Arabidopsis*. Science.

[25] To TK, Nakaminami K, Kim JM, Morosawa T, Ishida J, Tanaka M, Yokoyama S, Shinozaki K, Seki M (2011). *Arabidopsis HDA6* is required for freezing tolerance. Biochem Biophys Res Commun.

[26] Bertrand C, Bergounioux C, Domenichini S, Delarue M, Zhou DX (2003). Arabidopsis histone acetyltransferase AtGCN5 regulates the floral meristem activity through the *WUSCHEL/AGAMOUS* pathway. J Biol Chem.

[27] Pontvianne F, Blevins T, Pikaard CS (2010). Arabidopsis histone lysine methyltransferases. Adv Bot Res.

[28] Lu ZH, Huang XL, Ouyang YD, Yao JL (2013). Genome-wide identification, phylogenetic and co-expression analysis of *OsSET* gene family in rice. PLoS One.

[29] Qian YX, Xi YL, Cheng BJ, Zhu SW (2014). Genome-wide identification and expression profiling of DNA methyltransferase gene family in maize. Plant Cell Rep.

[30] Xu J, Xu H, Liu Y, Wang X, Xu Q, Deng X (2015). Genome-wide identification of sweet orange (*Citrus sinensis*) histone modification gene families and their expression analysis during the fruit development and fruit-blue mold infection process. Front Plant Sci.

[31] Fan S, Wang J, Lei C, Gao C, Yang Y, Li Y, An N, Zhang D, Han M (2018). Identification and characterization of histone modification gene family reveal their critical responses to flower induction in apple. BMC Plant Biol.

[32] Gu T, Han Y, Huang R, Mcavoy RJ, Li Y (2016). Identification and characterization of histone lysine methylation modifiers in *Fragaria vesca*. Sci Rep UK.

[33] Ahmad B, Zhang S, Yao J, Rahman MU, Hanif M, Zhu Y, Wang X (2019). Genomic organization of the B3-domain transcription factor family in grapevine (*Vitis vinifera* L.) and expression during seed development in seedless and seeded cultivars. Int J Mol Sci.

[34] Ahmad B, Yao J, Zhang S, Li X, Zhang X, Yadav V, Wang X (2020). Genome-wide characterization and expression profiling of GASA genes during different stages of seed development in grapevine (*Vitis vinifera* L.) predict their involvement in seed development. Int J Mol Sci.

[35] Holub EB (2001). The arms race is ancient history in *Arabidopsis*, the wildflower. Nat Rev Genet.

[36] Lee TH, Tang HB, Wang XY, Paterson AH (2013). PGDD: a database of gene and genome duplication in plants. Nucleic Acids Res.

[37] Siltberg J, Liberles DA (2002). A simple covarion-based approach to analyse nucleotide substitution rates. J Evol Biol.

[38] Joung JG, Corbett AM, Fellman SM, Tieman DM, Klee HJ, Giovannoni JJ, Fei ZJ (2009). Plant MetGenMAP: an integrative analysis system for plant systems biology. Plant Physiol.

[39] Wang L, Hu X, Jiao C, Li Z, Fei Z, Yan X, Liu C, Wang Y, Wang X (2016). Transcriptome analyses of seed development in grape hybrids reveals a possible mechanism influencing seed size. BMC Genomics.

[40] Howe EA, Sinha R, Schlauch D, Quackenbush J (2011). RNA-Seq analysis in MeV. Bioinformatics.

[41] Wang L, Yin XJ, Cheng CX, Wang H, Guo RR, Xu XZ, Zhao J, Zheng Y, Wang XP (2015). Evolutionary and expression analysis of a MADS-box gene superfamily involved in ovule development of seeded and seedless grapevines. Mol Gen Genomics.

[42] Morgan CC, Loughran NB, Walsh TA, Harrison AJ, O'Connell MJ (2010). Positive selection neighboring functionally essential sites and disease-implicated regions of mammalian reproductive proteins. BMC Evol Biol.

[43] Nelissen H, Boccardi TM, Himanen K, Van Lijsebettens M (2007). Impact of core histone modifications on transcriptional regulation and plant growth. Crit Rev Plant Sci.

[44] Shen W-H, Xu L (2009). Chromatin remodeling in stem cell maintenance in *Arabidopsis thaliana*. Mol Plant.

[45] Aquea F, Vega A, Timmermann T, Josefina PM, Arce-Johnson P (2011). Genome-wide analysis of the SET DOMAIN GROUP family in grapevine. Plant Cell Rep.

[46] Aquea F, Timmermann T, Arcejohnson P (2010). Analysis of histone acetyltransferase and deacetylase families of *Vitis vinifera*. Plant Physiol Biochem.

[47] Cannon S, Mitra A, Baumgarten A, Yong N, May G (2004). The roles of segmental and tandem gene duplication in the evolution of large gene families in *Arabidopsis thaliana*. BMC Plant Biol.

[48] Jaillon O, Aury J, Noel B, Policriti A, Clepet C, Casagrande A, Choisne N, Aubourg S, Vitulo N, Jubin C (2007). The grapevine genome sequence suggests ancestral hexaploidization in major angiosperm phyla. Nature.

[49] Xia F, Sun T, Yang S, Wang X, Chao J, Li X, Hu J, Cui M, Liu G, Wang D (2019). Insight into the B3 transcription factor superfamily and expression profiling of *B3* genes in axillary buds after topping in tobacco (*Nicotiana tabacum* L.). Genes.

[50] Lyons E, Pedersen B, Kane J, Alam M, Ming R, Tang HB, Wang XY, Bowers J, Paterson A, Lisch D (2008). Finding and comparing syntenic regions among Arabidopsis and the outgroups papaya, poplar, and grape: CoGe with rosids. Plant Physiol.

[51] Zhong SL, Fei ZJ, Chen YR, Zheng Y, Huang MY, Vrebalov J, McQuinn R, Gapper N, Liu B, Xiang J (2013). Single-base resolution methylomes of tomato fruit development reveal epigenome modifications associated with ripening. Nat Biotechnol.

[52] Li C, Xu J, Li J, Li Q, Yang H (2014). Involvement of Arabidopsis histone acetyltransferase *HAC* family genes in the ethylene signaling pathway. Plant Cell Physiol.

[53] Huang L, Sun Q, Qin F, Li C, Zhao Y, Zhou D (2007). Down-regulation of a *SILENT INFORMATION REGULATOR2*-related histone deacetylase gene, *OsSRT1*, induces DNA fragmentation and cell death in rice. Plant Physiol.

[54] Wang C, Gao F, Wu J, Dai J, Wei C, Li Y (2010). Arabidopsis putative deacetylase AtSRT2 regulates basal defense by suppressing *PAD4*, *EDS5* and *SID2* expression. Plant Cell Physiol.

