# Supplementary figures and images for: Bioinformatics and expression analysis of histone modification genes in grapevine predict their involvement in seed development, powdery mildew resistance, and hormonal signaling
Source: BMC Plant Biol. 2020 Sep 4;20:412. doi: 10.1186/s12870-020-02618-7 (PMC7473812; doi:10.1186/s12870-020-02618-7)

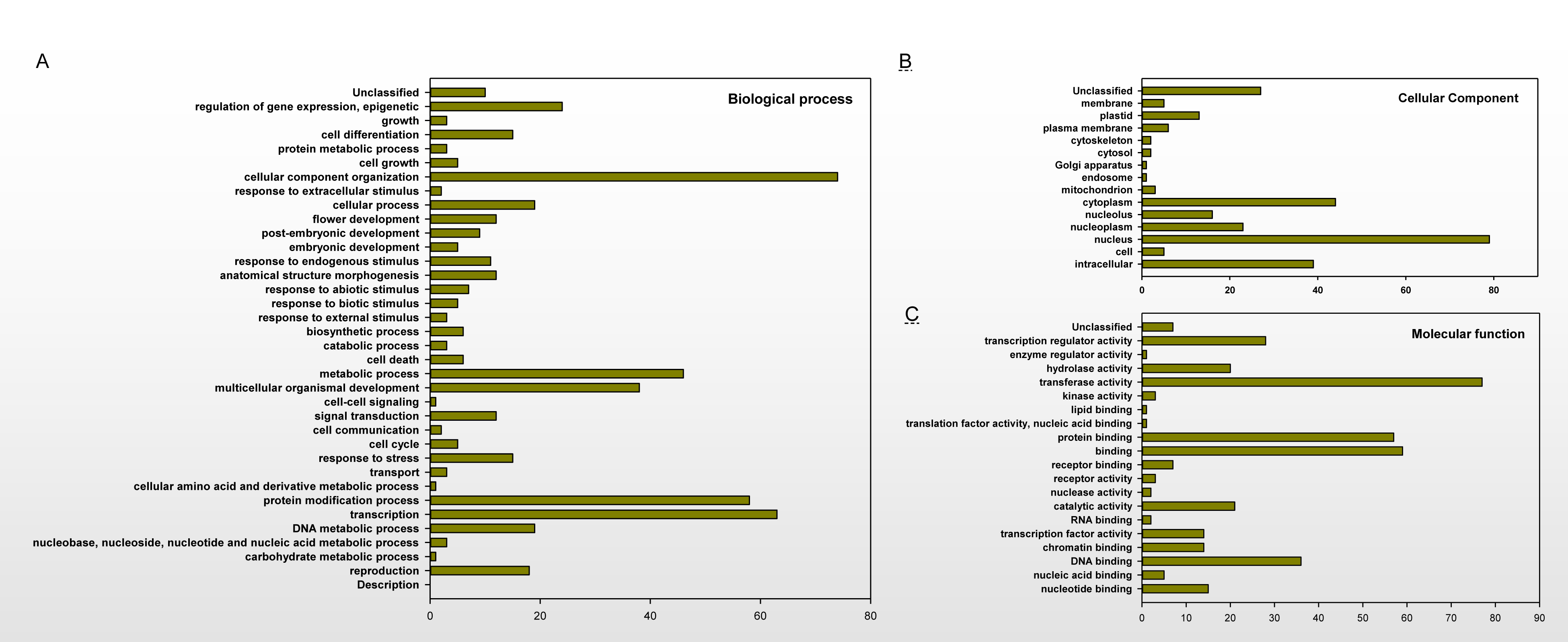

Supplement: Supplementary file 2 — Additional file 2: Figure S1. Gene ontology analysis of VvHMs. [file 12870_2020_2618_MOESM2_ESM.tif]
